# Transgenic Mouse Models of Alzheimer’s Disease: An Integrative Analysis

**DOI:** 10.3390/ijms23105404

**Published:** 2022-05-12

**Authors:** Raquel Sanchez-Varo, Marina Mejias-Ortega, Juan Jose Fernandez-Valenzuela, Cristina Nuñez-Diaz, Laura Caceres-Palomo, Laura Vegas-Gomez, Elisabeth Sanchez-Mejias, Laura Trujillo-Estrada, Juan Antonio Garcia-Leon, Ines Moreno-Gonzalez, Marisa Vizuete, Javier Vitorica, David Baglietto-Vargas, Antonia Gutierrez

**Affiliations:** 1 Departamento Biologia Celular, Genetica y Fisiologia, Instituto de Investigacion Biomedica de Malaga-IBIMA, Facultad de Ciencias, Universidad de Malaga, 29071 Malaga, Spain; raquelsv@uma.es (R.S.-V.); marinamejias@uma.es (M.M.-O.); juanjofv@uma.es (J.J.F.-V.); cristinand@uma.es (C.N.-D.); lauracaceres@uma.es (L.C.-P.); lauravegas@uma.es (L.V.-G.); elisanchez@uma.es (E.S.-M.); laura_trujillo@uma.es (L.T.-E.); jgarleon@uma.es (J.A.G.-L.); inesmoreno@uma.es (I.M.-G.); 2 Centro de Investigacion Biomedica en Red Sobre Enfermedades Neurodegenerativas (CIBERNED), 28031 Madrid, Spain; mvizuete@us.es (M.V.); vitorica@us.es (J.V.); 3 Departamento Fisiologia Humana, Histologia Humana, Anatomia Patologica y Educacion Fisica y Deportiva, Facultad de Medicina, Universidad de Malaga, 29071 Malaga, Spain; 4 Department of Neurology, McGovern Medical School, The University of Texas Health Science Center at Houston, Houston, TX 77030, USA; 5 Departamento Bioquimica y Biologia Molecular, Facultad de Farmacia, Universidad de Sevilla, Instituto de Biomedicina de Sevilla (IBIS)-Hospital Universitario Virgen del Rocio/CSIC, 41012 Seville, Spain

**Keywords:** Alzheimer’s disease, transgenic mice, amyloid, tau, microglia, astrocytes, oligodendrocytes, neurodegeneration

## Abstract

Alzheimer’s disease (AD) constitutes the most prominent form of dementia among elderly individuals worldwide. Disease modeling using murine transgenic mice was first initiated thanks to the discovery of heritable mutations in amyloid precursor protein (APP) and presenilins (PS) genes. However, due to the repeated failure of translational applications from animal models to human patients, along with the recent advances in genetic susceptibility and our current understanding on disease biology, these models have evolved over time in an attempt to better reproduce the complexity of this devastating disease and improve their applicability. In this review, we provide a comprehensive overview about the major pathological elements of human AD (plaques, tauopathy, synaptic damage, neuronal death, neuroinflammation and glial dysfunction), discussing the knowledge that available mouse models have provided about the mechanisms underlying human disease. Moreover, we highlight the pros and cons of current models, and the revolution offered by the concomitant use of transgenic mice and omics technologies that may lead to a more rapid improvement of the present modeling battery.

## 1. Introduction

In the last decades, murine models have become a powerful tool, not only to unravel the basic pathogenic mechanisms of human diseases, but also to identify therapeutic targets and perform preclinical assays. Given the inherent complexity of the central nervous system (CNS) and the need of reproducing behavioral/cognitive deficits, these animal models are even more valuable in the context of neurodegenerative disorders. Among all of them, Alzheimer’s disease (AD) constitutes the most prevalent type of dementia, affecting around 55 million people worldwide [1], and there is no cure for this medical, economic and social scourge [2].

The discovery of inheritable mutations directly responsible for the familial AD (FAD) cases, an early-onset form of the disease, enabled the creation of the first generation of transgenic (Tg) mouse models of this disease in the mid-1990s [3]. Initially, these Tg mice were based on the overexpression of mutated human amyloid precursor protein (APP) [4]. Thus, APP-based animals constituted the first approach to reproduce and analyze the histopathological progression of cerebral amyloid-β (Aβ) deposition, as well as different processes associated with amyloidosis, such as plaque formation, glial reaction, dystrophic neurites, synaptic damage, neuronal loss and memory impairment [4,5,6]. Given that APP-models were able to generate hyperphosphorylated tau (ptau), but not intraneuronal neurofibrillary tangles (NFTs), the incorporation of mutated *MAPT* gene allowed for investigating the tauopathy component of AD [7]. Although AD is not linked to tau mutations, mice co-expressing mutated *APP* and *MAPT* genes provided the first framework to unravel the interaction between both proteinopathies in vivo.

E4 allele of apolipoprotein E (APOE) was established early on as the main risk factor for the most common sporadic late-onset form of AD (sAD). Since then, APOE variants have been introduced in rodents to study the implication of this apolipoprotein on AD pathogenesis [8]. Similarly, the recent and invaluable information about sAD-risk factors yielded by genome-wide association studies (GWAS) enlarged a list that continues to expand with proteins related to the immune system, lipid metabolism and synaptic function, among others [9,10].

Indeed, the new set of Tg animals is currently providing an improved in vivo context to better replicate the mechanisms underlying sAD, including specific patterns of pathological progression or the interaction between AD and comorbidities [11]. However, there are still gaps to be filled. The MODEL-AD consortium [12] is facing the challenge of generating novel Tg lines to address the existing shortcomings in AD modelling, in order to accelerate the discovery of an effective therapeutic strategy for this disabling disorder. In this review, we provide an historical and functional overview about the past, present and future of AD-Tg murine lines, covering the landscape of approaches for each specific topic, including amyloidosis, tauopathy, neurodegeneration and the involvement of glial cells (microglia, astroglia and oligodendroglia).

## 2. Amyloid Pathology: A Long History from an Old Friend

### 2.1. APP-Based Models, the Beginning

The formation of extracellular amyloid deposits has been classically considered the main histopathological AD hallmark. These plaques composed of aggregated Aβ peptides (mostly Aβ40 and Aβ42 isoforms) are distributed throughout the brain parenchyma of patients [13,14]. This distinctive mark has been deeply studied in Tg APP-based murine models [4]. The first set of amyloidogenic mice emerged in the 1990s thanks to the progress of genetic editing techniques [7]. These models were based on the overexpression of the human *APP* gene bearing FAD mutations (Table 1). The most commonly used genetic variation is the Swedish mutation (Swe; K670N/M671L), which causes an increase in Aβ production [3,4]. These initial models have provided a valuable insight into understanding plaque formation as well as other AD features, such as cognitive impairment, synaptic damage and neuroinflammation [11].

Presenilin-1 (*PS1*) and presenilin-2 (*PS2*) genes encode for subunits of the γ-secretase, an enzymatic complex involved in APP processing and Aβ generation. Initially, the discovery of the role played by *PS* mutations in FAD led to the creation of novel monogenic models [15]. Tg mice expressing familiar mutations in either *PS1* or *PS2* presented an altered processing of murine APP, however, they failed at developing plaques [16], confirming that the expression of human *APP* is a must to reproduce Aβ deposition in mice. It is important to note that rodent Aβ differs in three amino acids with respect to the human protein [17], making it difficult to aggregate in the form of plaques. Then, a new generation of double Tg mice co-expressing mutations in human *APP* and *PS1* or *PS2* was generated. Among them, the most used bigenic lines are the APPswe/PS1dE9, APP751SL/PS1M146L and 5xFAD mice [4,18,19,20]. In general, these models develop an accelerated and more aggressive amyloidosis than the single APP-based models do, accompanied by an earlier appearance of the other classical cognitive and histopathological hallmarks (Table 1).

**Table 1:** Main monogenic and bigenic APP-based mouse lines.

APP-Based Monogenic Models	Mutations	Neuropathology	Onset Cognitive Impairment	References
PDAPP (line 109)	APP Indiana (V717F)	Aβ deposition (neuritic and diffuse) and gliosis by 6–9 months; synaptic loss	3 months	[3]
Tg2576	APP695 Swedish (KM670/671NL)	Aβ deposition (neuritic and diffuse) and gliosis by 11–13 months; synaptic loss	6–12 months	[21]
APP23	APP751 Swedish	Aβ deposition (neuritic and diffuse) and gliosis by 6 months; neuronal and synaptic loss	3 months	[22]
APP R1.40	APP Swedish	Aβ deposition (neuritic and diffuse) and gliosis by 14 months	Unknown	[23]
J20	APP Swedish, Indiana	Aβ deposition (neuritic and diffuse) and gliosis by 5–7 months; neuronal and synaptic loss	4 months	[24]
APP Dutch	APP751 Dutch E693Q	CAA from 22 to 24 months	Unknown	[25]
TgCRND8	APP695 Swedish, Indiana	Aβ deposition (neuritic and diffuse) and gliosis by 3 months; neuronal and synaptic loss	3 months	[26]
ArcAβ	APP695 Swedish, Arctic (E693G)	Aβ deposition and CAA from 9 months	6 months	[27]
Arc48	APP Swedish, Indiana, Artic	Aβ deposition (neuritic) and gliosis by 2–3 months	3–4 months	[28]
Tg-SwDI	APP770 Swedish, Dutch, Iowa (D694N)	CAA from 3 months and gliosis by 6 months	3 months	[29]
App NL-F KI	Humanized App Swedish, Iberian I716F	Aβ deposition (neuritic) and gliosis by 6 months; synaptic loss	18 months	[30]
App NL-G-F KI	Humanized App Swedish, Iberian, Arctic	Aβ deposition (neuritic) and gliosis by 2 months	6 months	[30]
hAβ-KI	Humanized Aβ sequence	Insoluble Aβ increase (without extracellular deposition) and cytokine alterations; synaptic loss	10–14 months	[31]
PS/APP	APP Swedish; PSEN1 M146L (A > C)	Aβ deposition (neuritic and diffuse) and gliosis by 6 months; neuronal loss	3–6 months	[32]
APP751SL/PS1M146L	APP751 Swedish, London (V717I); PSEN1 M146L	Aβ deposition (neuritic and diffuse) and gliosis by 3 moths; neuronal and synaptic loss	6 months	[19]
APP751SL/PS1-KI	APP751 Swedish, London; PSEN1 M233T, PSEN1 L235P	Aβ deposition and gliosis by 2.5 months; neuronal and synaptic loss	6 months	[33]
APPswe/PS1dE9 (line 85)	APP Swedish; PSEN1 deltaE9	Aβ deposition (neuritic) and gliosis by 6–9 months; neuronal and synaptic loss	12 months	[34]
APP/PS1	APP Swedish; PSEN1 L166P	Aβ deposition and gliosis by 1.5 months; synaptic loss	7 months	[35]
5xFAD (B6SJL)	APP Swedish, Florida (I716V), London; PSEN1 M146L (A > C), PSEN1 L286V	Aβ deposition (neuritic and diffuse) by 2 months; neuronal and synaptic loss	4–5 months	[20]
PS2/APP	APP751 Swedish; PSEN2 Volga German N141I	Aβ deposition (neuritic and diffuse) and gliosis by 6 months	8 months	[36]

Comparison of key-factors from the main transgenic mouse models expressing APP, including the mutations, and the age of onset of histopathological and cognitive hallmarks. APP: amyloid precursor protein; Aβ: amyloid-beta; CAA: cerebral amyloid angiopathy; PS, PSEN: presenilin.

### 2.2. Amyloid Aggregation and Deposition: Modelling a Subset of Plaques

**Figure 1:**
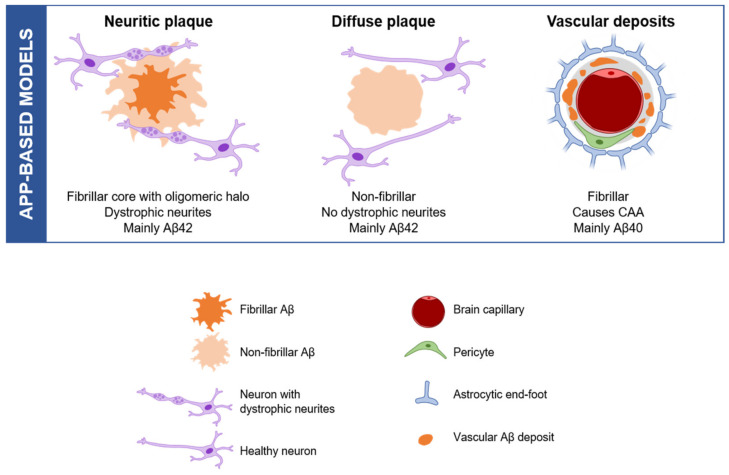
Amyloid plaque types in APP-based models. Neuritic plaques exhibit a fibrillar core encircled by a ring of oligomeric Aβ, and are surrounded by swollen neuronal projections, named dystrophic neurites. Conversely, diffuse plaques lack the fibrillar nucleus and do not display aberrant neuropile around them. Cerebral amyloid angiopathy (CAA) consists in vascular deposits of amyloid fibrils that accumulate within wall vessels of the brain. Parenchymal deposits are mainly formed by Aβ42, while vascular deposits are composed of Aβ40.

Aβ peptides are able to aggregate creating small soluble neurotoxic oligomers [37], which may also function as intermediates in the formation of more insoluble forms, the amyloid fibrils [38]. Several morphological types of amyloid plaques have been described within AD brains: fibrillar, classic, cotton wool, coarse-grained, etc. [14,39]. This variety of deposits can be classified into two main groups: neuritic and diffuse plaques. Neuritic plaques exhibit a fibrillar core surrounded by an oligomeric halo, together with dystrophic neurites (DNs), which are abnormally swollen neuronal processes (mainly axons and synaptic terminals). On the other hand, diffuse plaques are non-fibrillar and they have neither a core nor DNs surrounding them [40]. Aggregated Aβ peptides do not exclusively deposit in brain parenchyma, as they can also accumulate on blood vessels causing cerebral amyloid angiopathy (CAA). With respect to their composition, plaques mainly consist of Aβ42 isoform, while vascular deposits are formed by Aβ40 [41]. A subset of plaques is reproduced by different Tg APP models (Figure 1). For example, APPswe/PS1dE9, APP751SL/PS1M146L and 5xFAD mice develop fibrillar and neuritic plaques, whereas Tg-SweDI (containing *APP* with Swedish K670N/M671L and Dutch/Iowa E693Q/D694N mutations) display more diffuse and vascular Aβ deposits [19,29]. Current rodent lines reproduce the diversity of plaques described in AD brains to some extent, but the morphological variety of plaques is wider in human samples. Cotton wool plaques are mainly found in FAD patients with a deletion in *PS1* gene (PS1dE9); however, they are not present in APPswe/PS1dE9 Tg mice [34]. These plaques are immunoreactive for Aβ, but lack of a dense core or neuritic pathology [42]. Another example is the coarse-grained plaque, which has only been described in sAD cases [39]. These deposits exhibit an ill-defined border and multiple cores with Aβ-devoid pores, giving it a grainy appearance. DNs are often, but not always, present in this type of plaque. Overall, the complexity of amyloid deposition suggests that animal models able to better recapitulate this salient feature are still needed to uncover the biological and molecular pathways underlying this phenomenon.

### 2.3. Aggregation and Prion-like Spreading Mechanisms

Amyloidogenic animal models constitute a useful tool to study multiple pathological processes underlying this neurodegenerative disease [11]. For example, these lines have provided invaluable insight to determine the mechanisms of Aβ aggregation and spreading throughout the entire brain. In AD, misfolded Aβ has a higher tendency to aggregate, behaving as a self-propagating seed that induces the misfolding and aggregation of native Aβ [43,44]. In other words, Aβ propagates in a prion-like manner. For instance, the injection of brain-derived Aβ seeds from the cortical area of AD patients accelerated amyloid pathology in Tg mice [45,46,47]. Importantly, the increase of amyloidosis occurred not only at the site of injection, but also within synaptically connected brain regions. This result supports the idea of seeding-mediated Aβ propagation, and the involvement of axonal transport (either retrograde or anterograde) in the spreading [48]. Nevertheless, the propagation of Aβ seeds by passive diffusion cannot be ruled out [43]. Finally, several studies support that microglia play an important role in Aβ seeding and plaque formation [49,50,51].

### 2.4. Knock-In Mice to Knock-Out the Limitations

In spite of the progress achieved, the need for a shift in the modelling strategy became urgent due to continued failures at translating preclinical data obtained from FAD-based animals to AD patients [2]. In this regard, it is important to highlight some setbacks related to the current Tg lines: the level of *APP* expression is not genuine, since the transgene construction includes different promoters (Thy, PrP or PDGFB) to produce an intense neuronal expression. Hence, the overexpression of *APP* not only leads to an AD-like phenotype, but also produces artefacts including the overproduction of fragments other than Aβ [52], such as APP-CTFs, among others. Moreover, it has become evident that every single Tg model develops a specific phenotype depending on their unique construction (promoter, FAD mutations, the background of the strain, etc.) [4], hampering data interpretation and the comparison of results from different studies [11].

To minimize these limitations and facilitate successful preclinical to clinical translation, the developing and characterization of animal models able to better imitate the slower pace and progression of sAD, the most common disease form, is currently ongoing [53]. Similarly, Saito et al. [30] generated several strains of *App* knock-in (*App*-KI) mice with the aim of conducting the build-up of humanized murine-Aβ, eliminating the artifacts provoked by *APP* overexpression. This new generation of mice expresses humanized *App* at physiological levels, bearing FAD Swedish (NL) and Iberian (F) mutations (*App NL-F*), alone or in combination with the Artic mutation (*App NL-G-F*), which could allow gaining a deeper understanding about the downstream sequela of Aβ. Specifically, these models achieved reproducing Aβ42 overproduction and plaque deposition, synaptic loss, neuroinflammation and age-related memory impairment [4,30,54]. It is important to note that the plaques were more similar to those of humans, since the aggregates are constituted by Aβ42, followed by Aβ3(pE)-42. Therefore, *App*-KI mice might be a helpful preclinical AD model, but there are still pending limitations to be overcome.

Indeed, there is a growing need to design different AD models to identify effective therapeutic targets to be translated into successful clinical assays. With the aim of addressing this challenge, the US National Institute on Aging established a consortium in 2016, named Model Organism Development and Evaluation for Late-Onset Alzheimer’s disease (MODEL-AD) [12]. This consortium, led by Bruce Lamb (Indiana University) and Frank LaFerla (University of California at Irvine), seeks to create better models of sAD pathology [53]. In this regard, MODEL-AD has developed several humanized animal models, such as a hAβ-KI, Tau-KI lines and APOE4-KI mice (discussed later), displaying several aspects of the late-onset form of AD. For example, the hAβ-KI model exhibits age-dependent cognitive and synaptic deficits associated with changes in amyloid levels, astrocyte reactivity, the appearance of Periodic Acid-Schiff (PAS) granules and changes in gene expression [31]. Hopefully, these novel Tg lines may serve as valuable tools to investigate novel disease mechanisms and translating preclinical efficacy to clinical outcome.

## 3. Transgenic Models of Tauopathies to Unravel Alzheimer’s Disease

### 3.1. Tau Aggregation and Tauopathies

Tau belongs to the family of microtubule-associated proteins (MAPTs) and is mainly expressed in neurons. This protein fulfills a variety of functions, among which microtubular stabilization and axonal transport regulation stand out. In the adult human brain, there are six isoforms of tau ranging from 352 to 441 amino acids, generated by alternative splicing of exons 2, 3 and 10. The inclusion of exons 2 and 3 involves the insertion of amino acids at the N-terminal region (0N, 1N or 2N), 0N and 1N being the majority isoforms. The addition of exon 10 implies an extra microtubule-binding repeat domain (MBRD) within the C-terminus, giving rise to isoforms with four (4R) instead of three repeats (3R). It is important to highlight that in the adult human brain the 3R and 4R isoforms are similarly distributed, whereas mouse brain contains exclusively 4R tau [7].

Numerous posttranslational modifications are involved in the modulation of tau properties and activities, either under physiological or pathological conditions. To date, at least 85 specific sites of phosphorylation have been reported along this protein [55]. Abnormal hyperphosphorylation triggers the detachment of tau from microtubules, facilitating its misfolding and aggregation, and thus interfering with axonal transport. Eventually, these alterations lead to synaptic impairment and neuronal dysfunction [56]. Similarly, tauopathies are a group of neurodegenerative diseases characterized by the aggregation and accumulation of ptau, including AD. Primary tauopathies or frontotemporal lobar degeneration include frontotemporal dementia (FTD), progressive supranuclear palsy (PSP), corticobasal degeneration (CBD) and globular glial tauopathy (GGT). Further tauopathies comprise argyrophilic grain disease (AGD), primary age-related tauopathy (PART) and chronic traumatic encephalopathy (CTE) [57,58]. The 3R isoform is present in FTD, whereas 4R aggregates are found in PSP, CBD, AGD and GGT. Specifically, AD brains display tau aggregates with a mixed 3R/4R ratio (such as CTE and Down syndrome) [57], in addition to extracellular Aβ deposits. In AD brains, aggregated ptau accumulates in structures such as dystrophic neurites, neuropile threads and NFTs. Strikingly, brain ptau accumulation correlates more closely with cognitive decline than Aβ deposits do [59].

### 3.2. We Do What We Can: Generating Tau Pathology Using Tauopathy Models

Mutated human *MAPT* gene has been extensively used to reproduce tau pathology in murine models (Table 2). The first single Tg mouse model of tauopathy was the JNPL3 line expressing the P301L mutation (4R/0N) [60]. This specific mutation is associated with frontotemporal dementia with parkinsonism linked to chromosome 17 (FTDP-17). As early as 4.5 months, these animals develop NFTs and body inclusions, especially in the amygdala and spinal cord. Most of these mice display motor and behavior impairment by 10 months [61]. PS19 is another popular single Tg mouse, which overexpresses P301S tau mutation under the mouse prion promoter (PrP). This line shows ptau from 6 months and NFTs formation by 8 months in the hippocampus, amygdala and cortical areas, along with prominent pathology in the brain stem and spinal cord. In addition, these animals exhibit microgliosis from 3 months, synaptic dysfunction at 6 months, and neuronal loss by 8–12 months [62]. Furthermore, the model demonstrates learning and memory impairment as well as motor weakening and paralysis. Whereas P301S mutation causes inability of microtubule assembly, P301L mutation interferes with the rate of formation of paired helical filaments (PHFs) in vitro [63]. There are two additional mouse lines expressing P301L, whose expression is suppressed by doxycycline. The rTg4510 model expresses the P301L mutant human tau (4R0N), mainly in the forebrain. These mice show tangle-like inclusions by 4 months, together with extensive neuronal loss and brain atrophy [64]. The Thy-Tau22 mouse model expresses the human 4R1N tau with G272V and P301S mutations under a Thy1.2-promotor. This model presents early-onset tau pathology, including NFT-like inclusions, PHFs and ghost-tangles, along with microgliosis and reactive astrogliosis from 3 months. One specific advantage of these mice consists of the development of cognitive impairment by 10 months without displaying motor deficits [65].

As shown, mice expressing mutant tau provide an important tool for the study of tauopathies. Nevertheless, mutations in the gene encoding tau have not been associated with an increased risk of developing AD thus far. Moreover, these animals produce the endogenous tau protein, giving rise to pathology in brain areas which are less relevant for this type of dementia. Besides, they present an undesired combination of mutated human and murine tau protein. In this sense, htau is a mouse model that exclusively expresses the six isoforms of wild-type (WT) human tau. In these mice, ptau accumulation appears in neuronal soma and dendrites from 3 months. Remarkably, NFT-like pathology is detectable by 9 months displaying a spatiotemporal distribution similar to that of human brains [66].

To provide animal models able to better recapitulate the complexity of AD neuropathology, mutated *MAPT* has been combined with FAD mutations. For instance, APPswe-Tau mice were generated by crossing Tg2576 animals with P301L mice. The resulting model shows an amyloid pathology similar to the Tg2576 mouse, together with an increased tau pathology [61]. The 3xTg-AD mouse model bears mutations in three genes, *PS1* (M146V), *APP* (swe) and *MAPT* (P301L), which leads to the development of amyloid plaques at 6 months and tau pathology at 12 months, accompanied by deficits in learning and spatial memory [67,68]. More mixed models have been obtained by either silencing (knock-out, KO) or including other sAD risk-factor genes (discussed later), such as the triggering receptor expressed on myeloid cells 2 (*TREM2*) as in the PS19/Trem2-KO [69] and PS19/hTREM2 mice [70], respectively.

Even though animals containing non-mutated *MAPT* may reproduce tau-associated pathology of AD brains, spontaneous models of tau pathology are still needed in the field. This point is especially relevant for preclinical studies to bear fruit when translated into clinical trials. In regard to this, the *Mapt*-KI is a model of pathological dissemination of human tau bearing a humanized *Mapt* gene. The complete genomic sequence of the murine *Mapt* is replaced with the human *MAPT* gene. *Mapt*-KI mice exhibit higher expression of the 3R isoform, whereas WT mouse brain contains exclusively the 4R tau isoform. *Mapt*-KI mice were subsequently crossed with *App* knock-in mice, giving rise to the *App*/*Mapt*-KI model. These animals exhibit higher tau phosphorylation (no NTFs), Aβ pathology, DNs and neuroinflammation at 6 months, making it an interesting tool for studying the pathogenesis of tauopathies within an AD-context [71].

**Table 2:** Summary of the main transgenic mouse models of tauopathy used in AD field.

Model	Mutations	Neuropathology	Onset Cognitive Impairment	References
JNPL3	P301L mutation (4R/0N) under mouse PrP promoter	NFTs and body inclusions by 4.5 months	10 months	[60]
htau	Wild-type MAPT under tau promoter	NFTs by 9 months and neuronal loss	12 months	[66]
rTg4510	P301L mutation suppressed by doxycycline, under human CaMKIIα promoter	NFTs by 4 months, neuronal loss and brain atrophy	3–5 months	[64,72]
Thy-Tau22	Human (4R/1N) tau under Thy1.2-promotor	NFTs and astrogliosis by 3 months	10 months	[65]
PS19 line	P301S mutation (4R/1N), under mouse PrP promoter	NFTs by 8 months, gliosis and synaptic dysfunction	3–6 months	[62]
rTgTauEC	P301L mutation in the entorhinal cortex, under neuropsin promoter	NFTs, synaptic degeneration and neuron loss by 3 months	Unknown	[73]
APPswe-Tau	Tg2576 and Tau P301L mutation, under mouse PrP promoter	NFTs and neuronal loss by 6 months	Unknown	[61]
App/Mapt dKI	Humanized *App* (Swedish, Arctic and Iberian) mutations, and humanized *Mapt* gene under CAM kinase II promoter	ptau, Aβ pathology, dystrophic neurites and neuroinflammation by 6 months	Unknown	[71]
3xTg-AD	PS1 (M146V), APP (swe) and MAPT (P301L) mutations under mouse Thy1.2 promoter (APP, MAPT) and endogenous PSEN1 promoter	Aβ plaques at 9 months, NFTs at 12 months	3–6 months	[67]

APP: amyloid precursor protein; Aβ: amyloid-beta; PSEN: presenilin; ptau: hyperphosphorylated tau; NFTs: neurofibrillary tangles.

### 3.3. Seeding, Spreading and Progression of Tau Pathology: Can We Model It?

Tau pathology follows a specific spatiotemporal progression pattern in tauopathies. Braak and Braak stages describe how NFTs initially form in the transentorhinal region (stages I and II), progressively spreading to the hippocampus (stages III–IV) and the neocortical region (stages V–VI) along the course of AD [74,75,76]. Patients in the earliest stages are asymptomatic, whereas signs of memory impairment are present from stages III to IV. Finally, patients at later stages display advanced symptoms. An important limitation to modeling tau pathology is that most of the available tau Tg mice do not reproduce the characteristic progression pattern. The rTgTauEC mouse was generated to better imitate this particular event of AD pathology. This line expresses the P301L mutant tau specifically in the entorhinal cortex, facilitating the study of tau propagation and the spatiotemporal pattern of distribution over time [73]. Thus, human tau protein propagates from entorhinal cortex neurons. Then, tau aggregates follow a pattern of spreading through synaptically connected neurons, leading to a progressive neurodegeneration more similar to the human cases [73,77]. In the case of CTE, tau aggregates give rise to NFTs, neuropil threads and astrocytic tangles. Tau aggregates have a proclivity to arrange near small blood vessels, ventricles and subpial tissues. In fact, the staging of tau pathology in CTE has been described similarly to Braak stages. In stage I, NFTs are located in the depths of frontal cortical sulci; in stage II, NFTs are found in the cortex. In stage III, NFTs spread throughout the amygdala and hippocampal area, and finally, through the cortex and temporal lobe in stage IV [78]. Several animal models have been generated in an effort to mimic the CTE progression in mice [79].

The hierarchical progression pattern of pathological tau within the brain may be explained by its prion-like nature, as suggested for Aβ spreading. The formation and accumulation of misfolded aggregates of tau follows a seeding-nucleation model. A small and normally soluble misfolded oligomer acts as a nucleus to propagate misfolding by recruiting the native proteins into the polymers. Introduction of a preformed seed can accelerate protein misfolding by attenuating the nucleation phase [80]. The process of seeded/nucleated polymerization has been reproduced in vitro, and is thought to be responsible for the spreading of the pathology in an infectious manner in prion diseases. Importantly, recent results have shown experimental evidence for prion-like mechanisms of pathological spreading of misfolded proteins associated with various diseases, including AD [43,81,82,83]. The prion-like spreading may constitute a molecular explanation for the progress of pathological abnormalities in tauopathies in a stereotypical and predictable manner across anatomical connections, usually starting from a circumscribed area and spreading all over the brain [73,84].

Several experiments have characterized the debated prion-like features of tau. Intracerebral injection of AD homogenates containing tau aggregates induced the conversion of native tau into misfolded aggregated filaments in mice [85,86]. Both, the transmission and induction of tau pathology depend on the original source of tau aggregates, indicating a conformation or strain-dependent transmission [87,88]. In addition, cerebral tau deposition was found in Tg mice that received intraperitoneal administration of brain homogenates containing tau seeds [89], suggesting that peripheral tau aggregates might modify the progression of brain pathology. In fact, tau aggregates can be found in many organs and biological fluids from AD patients as a soluble protein [90]. Therefore, data strongly suggest that seeding may be a responsible actor in the spreading of misfolded conformations between neuroanatomically connected brain regions [91].

Furthermore, there is increasing recognition from studies with tau-based mouse models indicating that microglial cells may be actively engaged in tau phagocytosis and dissemination across the brain as well [92,93]. For instance, Asai et al. [92] showed that microglial depletion in PS19 mice lead to tau spreading suppression, and proposed that these glial cells are able to take up and secrete tau seeds via exosomes. In this sense, a positron emission topography (PET) imaging-based study of aged and AD individuals has provided more evidence suggesting that activated microglia are involved in tau pathology propagation following the Braak spreading pattern [94].

Overall, current models of tau-related neuropathology recapitulate some of the major events observed in human AD brains. The main drawback is the need of using mutated *MAPT* gene (characteristic of other types of dementia) to model this disorder, even though AD patients do not show any tau-related mutation. Another important concern is the necessity of expressing high levels of the humanized gene, which are not representative of the constitutive expression, except for KI mice. Besides, the distinctive propagation pattern of tau aggregates limits a proper modeling, being a pivotal step of the disease development not observed in most models, with the exception of the mentioned rTgTauEC mouse. Finally, these animals constitute a useful tool for analyzing neurodegenerative disorders based on a sole misfolding protein. However, AD is mainly characterized by the accumulation of Aβ, forcing the generation of multi-transgenic mice in order to reproduce plaques and tangles, together with other hallmarks such as neuroinflammation and neuronal loss. It is also desirable to achieve a sequence of pathologic events matching with the timeline observed in humans. Therefore, novel tau-related models are needed for a more accurate study of AD and other tauopathies, including those bearing humanized (non-mutated) *Mapt* genes and KI animals, probably in combination with other transgenes so as to better mimic the spatiotemporal pathological phenomena of this neurodegenerative disease.

## 4. Modeling Synaptic Dysfunction and Neurodegeneration

Synaptic pathology is an early event in AD progression, being the feature that best correlates with clinical symptoms in patients [95]. In fact, synaptic alterations occur at the initial disease stages, prior to synapse loss and neuronal degeneration [96]. According to the amyloid hypothesis, Aβ triggers tau hyperphosphorylation and aggregation [97,98] contributing to synaptic dysfunction and neuronal death. For decades, the neurotoxic effects of Aβ and tau were established separately, but there is growing evidence showing that Aβ-induced neurodegeneration is mediated by tau [99,100,101]. Synaptic damage induced by either Aβ, tau or both, can be studied in vitro with neuronal culture and organotypic brain slices, but also in vivo using Tg mouse models.

### 4.1. Aβ Role on Synaptic Impairment

Aβ peptide has been proposed to exert a profound effect over synaptic transmission and intracellular signaling cascades of excitatory neurons. Compelling evidence points to soluble Aβ oligomers as the major neurotoxic species [37]. These Aβ forms act on multiple synaptic targets including N-methyl-D-aspartate (NMDA) and α7-nicotinic acetylcholine (α7-nACh) receptors, contributing to synapse dysfunction and loss [102,103]. Although the effects of Aβ over synapses have been mainly studied in cell culture, they are also reproduced by AD mouse models (Table 1). In this sense, muscarinic regulation of γ-amino-butyric acid (GABA) transmission has been shown to be impaired in an APP model, probably due to the Aβ interference with mAChR (muscarinic acetyl-choline receptors) [104]. In the APPSL/PS1M146L model, both synaptic and presynaptic vesicular densities are inversely correlated to plaque distance, and affected by the oligomeric halo from very early pathological stages [105]. In the APPswe/PS1dE9 model, synaptic depotentiation occurs before Aβ plaque deposition in the motor cortex and might be induced by soluble Aβ oligomers [106].

The presence of DNs constitutes another relevant AD hallmark pointing to the role of Aβ in synaptic damage and neurodegeneration. DNs are mostly abnormal swollen axons and presynaptic terminals, filled with autophagic vesicular content, indicating a disrupted axonal transport and synaptic dysfunction [107,108]. These aberrant neuronal processes are intimately associated with neuritic plaques, usually well reproduced by amyloidogenic models such as 5xFAD [109,110], APP23 [22], TgCRND8 [111], Tg2576 [111] and APP751SL/PS1M146L [19,108,112]. Importantly, periplaque dystrophies have been shown to be involved in Aβ generation, leading to a vicious amyloidogenic cycle [108,109,113,114].

### 4.2. Synaptic Pathology in Tau Mice

Tau hyperphosphorylation and aggregation are associated with impaired long-term potentiation (LTP) and short-term synaptic plasticity. Accordingly, synaptic dysfunction has been described in Tg tau mice [65,115]. Numerous models expressing either human WT tau (htau and triple Tg PLB1), mutant tau (including P301L, K257T/P301S, TauRDΔ280 expressing pro-aggregated 4R fragment K18) or a model of genetically-induced tau hyperphosphorylation (using the PP2A inhibitor CIP2A), show LTP alterations correlating with an increase in ptau and aggregation [115,116,117,118,119,120]. Besides, tau can mediate Aβ-induced cognitive deficits by modulating a non-receptor tyrosine Fyn kinase [121,122]. Synaptic strength depends on NMDA receptor activation which is regulated by PSD95-Fyn-NMDA receptor complex at the postsynaptic site [123]. Tau hyperphosphorylation affects the formation of this complex that will, in turn, attenuate synaptic function. In fact, tau pathology correlates better than amyloidosis with the degree of cognitive decline in AD patients [124]. Furthermore, once NFTs are formed, Aβ clearance cannot ameliorate the behavioral deficits [125]. However, tau removal improves cognitive function even though Aβ burden remained constant or increased [126,127,128]. Recent investigations further strengthened the hypothesis that neuritic plaques trigger the pathological conversion of tau in an AD mouse model [129]. Notably, aberrant neuropile in the form of dystrophic axons/presynaptic terminals are not found among single tau models, highlighting the pivotal role of Aβ in the generation of this histopathological hallmark prior to neuronal death.

### 4.3. Neurodegeneration in AD Mouse Models

Neuronal loss is a key feature of AD reported in several brain areas [130], such as the hippocampal CA1 region [131] and the entorhinal cortex [132]. Neurodegeneration initiates at preclinical stages, correlating with the severity of memory deficits [133]. The most affected, and thus the most studied neuronal populations in AD are pyramidal cells, inhibitory interneurons expressing GABA and cholinergic neurons. Neurochemical analysis has enabled the establishment of a neurodegenerative pattern. In fact, cholinergic markers and pyramidal cell number correlate with the severity of dementia [134]. The loss of cholinergic innervation to the cortex occurs at an early stage, whereas the loss of GABAergic neurons seems to occur at later points of the disease.

Even though most mouse models exhibit the main histopathological AD hallmarks (Aβ deposition and ptau accumulation), many of them fail at reproducing substantial neuronal loss [135]. The amyloid hypothesis postulates that Aβ accumulation in the brain is the primary cause of AD [124]. However, the mechanisms by which this peptide induces neuronal death is not yet fully understood [136]. It has been proposed that Aβ may trigger either mitochondrial dysfunction [137,138], endoplasmic reticulum stress [139], apoptosis [140] or in combination. In addition, intraneuronal Aβ-accumulation is an early event in the human AD brain [141] preceding amyloid plaque pathology [142] that has been linked to pyramidal neuron loss in some AD Tg models. Conversely, in contrast to APP/Aβ models, most tau mice exhibit age-dependent neurodegeneration along with the synaptic and cognitive deficits, suggesting that either misfolded, aggregated or both, tau is directly neurotoxic. Although tau mice are models of pure tauopathies [143] and have some evident shortcomings (as mentioned above), they serve as valuable tools to understand the basics of tau toxicity in AD.

#### 4.3.1. Cholinergic Degeneration

The loss of cholinergic neurons significantly contributes to the early cognitive decline of AD [144]. The nucleus basal of Meynert (nbM) is the main source of cholinergic innervation to the cerebral cortex [145] and display a profound neurodegeneration in patients [146]. In fact, the prevailing therapy for AD is based on the cholinergic hypothesis and the restoration of cholinergic function through cholinesterase inhibitors. These drugs (donepezil, rivastigmine, and galantamine) have been shown to induce symptomatic improvement in AD patients [144]. Unfortunately, the therapeutical effect of these treatments is reduced to a short-time window due to the spreading of the neurodegeneration to other cerebral regions, among other reasons.

Most of the FAD-based models exhibit degeneration of cortical and hippocampal cholinergic fibers [147,148,149] accompanied by memory deficits. Thus, PDAPP [150] and APP23 [148] showed an alteration of cholinergic fibers. However, these changes are not associated with a loss of cholinergic neurons in the basal forebrain, as occurs in AD patients. Nevertheless, some authors have described a cholinergic neurodegeneration in the medial septum of APP23 at 12–14 months of age [151]. Choline acetyl transferase (ChAT)-positive neurons were reduced in the medial septum of 5xFAD mice [152] and in motor nuclei from APP/PS-KI mice [153]. Moreover, the number of nbM cholinergic neurons was reported to be significantly decreased in 12-month-old Tg-SweDI mice [154], and in the model APPSL at 6 months of age [155].

Regarding tau-mediated cholinergic neurodegeneration, previous post-mortem studies have shown that the loss of cortical cholinergic innervation is associated with the presence of NFTs in the nbM [156]. Hyperphosphorylated tau, in the form of NFTs and pretangles, has been found specifically in the cholinergic neurons of the basal forebrain in cognitively normal elderly subjects and patients with mild cognitive impairment (MCI), and correlates significantly with performance in memory tasks [157]. Thus, basal forebrain cholinergic neurons are among those most susceptible to neurofibrillary degeneration and NFT formation [156]. Similarly, some tau models reproduce this cholinergic vulnerability. Thy-Tau22 mouse model displayed a reduction (28%) in the number of cholinergic neurons in the medial septum [158]. Moreover, a loss of cholinergic neurons has been reported in the basal forebrain of L1 mice (expressing truncated tau). Animals at 3, 6 and 9 months of age show a clear reduction in the number of ChAT neurons (30–60%) in several basal forebrain regions with a decrease in acetylcholinesterase staining in the cortex and the hippocampus [159]. Therefore, the cholinergic affectation described in AD patients is reproduced, at least in part, by both *APP* and *MAPT* Tg mouse models.

#### 4.3.2. GABAergic Neurodegeneration

Pyramidal network activity is coordinated by GABA interneurons which are often classified according to their immunoreactivity against calcium-binding proteins, such as parvalbumin (PV), somatostatin (SOM) or calretinin (CR) [160]. These subpopulations are affected differentially among distinct brain regions. In this sense, a decrease of PV-interneurons has been described in the hippocampus [161] and perirhinal cortex [162] of AD patients, while a preferential vulnerability of somatostatin (SOM)- and CR-cells was reported in the piriform cortex [163]. Specifically, SOM interneurons are severely affected in human AD brains [164].

In fact, hippocampal SOM dysfunction, as a result of decreased cholinergic input to these cells, resulted in memory impairment in amyloidogenic mice [165]. In this regard, we have described significant reductions of somatostatin/neuropeptide Y (SOM/NPY) interneurons in the hippocampus [166], subiculum [112] and entorhinal cortex [167] of the APP751SL/PS1M146L mouse model at early ages (4–6 months). Moreover, we also described a loss of CR interneurons starting at 4 months of age in this same model [168]. SOM- and NPY-interneurons were also reduced in the hippocampus of TgCRND8 mice [169,170]. As described above, the SOM-positive subpopulation is particularly affected in AD patients [171], and the loss/dysfunction of SOM-positive neurons is recurrent in models with Aβ plaques. Conversely, the status of GABAergic interneurons has barely been addressed in tau models in comparison to amyloidogenic mice. JNPL3 (BL6) mice displayed a reduction in hippocampal GABAergic interneurons (PV and SOM) [119]. Some other tau models have shown a GABAergic synaptic disruption without changes in the neuronal density. This is the case of rTg4510 mice [172] or the VLW line (G272V, P301L and R406W mutations) [173].

Reports concerning changes in PV interneurons are not without controversy. Thus, some models display PV loss within the hippocampus [170,174], while an increase [175], and even no change in the number of these interneurons has been described [169] in others. The same discordance has been observed in AD patients, with reports ranging from substantial loss to unchanged, or even an increased number of these cells [163,176,177]. We have previously reported that PV interneurons are preserved in the amyloidogenic APP751SL/PS1M146L model [112,162,166,167], suggesting that this specific GABAergic subpopulation is resistant to Aβ. On the other hand, studies in AD brains have found that regions early affected by tau pathology develop a significant loss of PV neurons [177,178]. Though the cause of PV neuronal death in AD remains to be elucidated, these differences between APP/PS1 model and AD brains point to a key role of ptau over this population. In fact, the accumulation of pSer262 and pThr205 tau has been recently reported within these cells [179], and the loss of PV-positive neurons has been described in several tau models [119,180].

#### 4.3.3. Pyramidal Neurodegeneration: The Achilles’ Heel of FAD Models

A significant decrease in pyramidal neuron density has been described in the subiculum and CA1 hippocampal region from AD patients [181], the layer II of entorhinal cortex [132] or the olfactory bulb [182]. Atrophy was also present within the basal ganglia, probably due to the loss of pyramidal cells [183]. However, among FAD-based models, only a few reproduce a substantial degree of neuronal loss. APP23 was the first Tg-AD mouse showing neuronal loss. The number of hippocampal CA1 pyramidal cells decreased 14–25% by 14–18 months of age [184]. In 5xFAD mice, stereological quantification confirmed a significant loss of cortical neurons at 9 and 12 months of age [185].

However, no cell death was observed in the hippocampus despite considerable APP expression. This lack of neurodegeneration may be due to the absence of intraneuronal Aβ accumulation, suggesting a correlation between these two processes [185]. Intraneuronal Aβ accumulation has been described in several mouse models, such as APP23 [186], APP751SL/PS1M146L [112] or 5xFAD [20], among others. For instance, pyramidal loss associated with intraneuronal Aβ has been reported in cortical layer 5 and subiculum of 5xFAD mice [20]. In the TgCRND8 model, a decreased volume and neuronal count was shown in CA1 [187]. Although intraneuronal Aβ accumulation was not detected in this region, Aβ was found within axons projecting from CA1 in organotypic hippocampal slice cultures from TgCRND8 mice [188]. The mouse model APPSL/PS1M146L displayed 35% of CA1 pyramidal neuron loss by 17 months of age [189]. Finally, the APP751SL/PS1-KI mice present a 30% of CA1 neuron loss at 6 months of age [190].

There are fewer reports about this topic concerning tauopathy models. Remarkably, some of them develop severe brain atrophy presumably due to neurodegeneration. For instance, rTg4510 mice lose up to 40% of gross brain weight and >65% of CA1 pyramidal neurons by 5.5 months of age [64,72,191]. Significant forebrain atrophy with loss of cortical and hippocampal volumes is also observed in the P301S tau model (PS19) at advanced ages [62]. Even the htau model overexpressing wild-type human *MAPT* develops neuronal loss, although only at late ages (17 months) [192].

### 4.4. Modeling Amyloid-Tau Synergy in Neurodegeneration

Although tau pathology correlates better with synaptic/cognitive deficits than Aβ, it is obvious that tauopathy models do not recapitulate amyloid deposition. Given that AD patients present both histopathological hallmarks, APP (or double APP/PS1) Tg mice were crossed with tau models [193] to generate a more accurate approach to this disease. Multiple studies have demonstrated that Aβ and tau interact to promote cognitive decline and neurodegeneration in mouse models [113]. Interestingly, behavioral phenotypes occur earlier and more severe in several APP x tau crosses than in their single-Tg siblings [194,195]. APP/tau mice showed a significant reduction in the number of neurons within CA1 and the entorhinal cortex [196]. Crossing the 5xFAD model with PS19 tau mice [62] resulted in a clear aggravation of the tau pathology with a robust hippocampal atrophy and severe loss of neurons in the CA1 layer [197]. Furthermore, 3xTg-AD mouse showed interneuron and cholinergic cell loss [152,180,198]. The number of ChAT-positive neurons was decreased in the basal forebrain accompanied by a reduction in the cholinergic fibers in cortical and hippocampal areas [198,199], and 33% of CR and 52% of PV-positive cells were lost in the CA1 hippocampal layer at 18 months [180]. Conversely, the double-KI model generated by crossing the humanized *Mapt*-KI with the *App*-KI model lacked neurodegeneration, even though it showed an increase in ptau levels compared to the single htau-KI [71].

Overall, the observations support the idea that Aβ and tau act together, and that Aβ is upstream of tau [98,200]. Among the putative mechanisms underlying Aβ-tau synergy, a direct interaction between both proteins has been proposed [101]. Several studies have reported that pathological Aβ and tau aggregates can colocalize in neurons and synaptic terminals in both human post-mortem tissue and 3xTg-AD mice, with an increased interaction in later disease stages [201]. Nevertheless, a recent quantitative array tomography study of human post-mortem tissue, as well as APP/PS1-rTg21221 mice, reported that synapses were positive for both Aβ and tau in less than 0.02% of cases [202]. Otherwise, Aβ and tau could interact indirectly through their impact on neuronal physiology and glial activation. The generation of appropriate preclinical models reproducing synaptic and neuronal loss mediated by both Aβ and tau is an essential goal for the identification of disease mechanisms as well as for the development of potential treatments.

## 5. Microglial Response in Transgenic Mice of Alzheimer’s Disease

Dysregulated innate immune responses in the brain have emerged to be critical in many neurodegenerative diseases, including AD [203,204]. Microglia are the primary resident immune cells of the brain which derived from erythromyeloid progenitors in the yolk sac. These glial cells perform multiple physiological functions, which are essential for brain development and adult brain homeostasis maintenance, such as surveillance, programmed cell death, clearance of apoptotic newborn neurons, synaptic pruning and neuronal plasticity, among others [205,206]. In addition to Aβ and tau pathologies, dysfunctional microglial response has been postulated as a driver for AD onset and development, especially since the identification by GWAS of several genetic AD-risk factors related to microglial functions (such as *CR1*, *CLU*, *MEF2C*, *EPHA1*, *TREM2*, *CD33*, *ABCA7* and *MS4A*), which constituted a decisive turning point in AD research [207]. Therefore, new strategies are constantly arising in the field of modeling microglial response: mutations or deletions in microglial genes linked to AD-risk are introduced in the classic AD models, the pharmacological or conditional genetic microglial depletion [208], and the inhibition/promotion of inflammatory state in these immune cells, among others [209,210,211].

### 5.1. Microglial Phenotypes in Classic Models of Amyloidosis and Tauopathy

Classically, microglial cells were categorized following a binomial model, depending on their activation status. Under physiological conditions, microglia usually manifest the homeostatic state, carrying out the above-mentioned non-inflammatory functions, crucial for CNS development. At the morphological level, homeostatic microglia display a compact and small cell body with many thin ramifications, however, under pathological conditions, microglia adopt a reactive profile showing enlarged cell body (hypertrophy), together with fewer and thicker branches displaying an amoeboid morphology [212,213].

**Figure 2:**
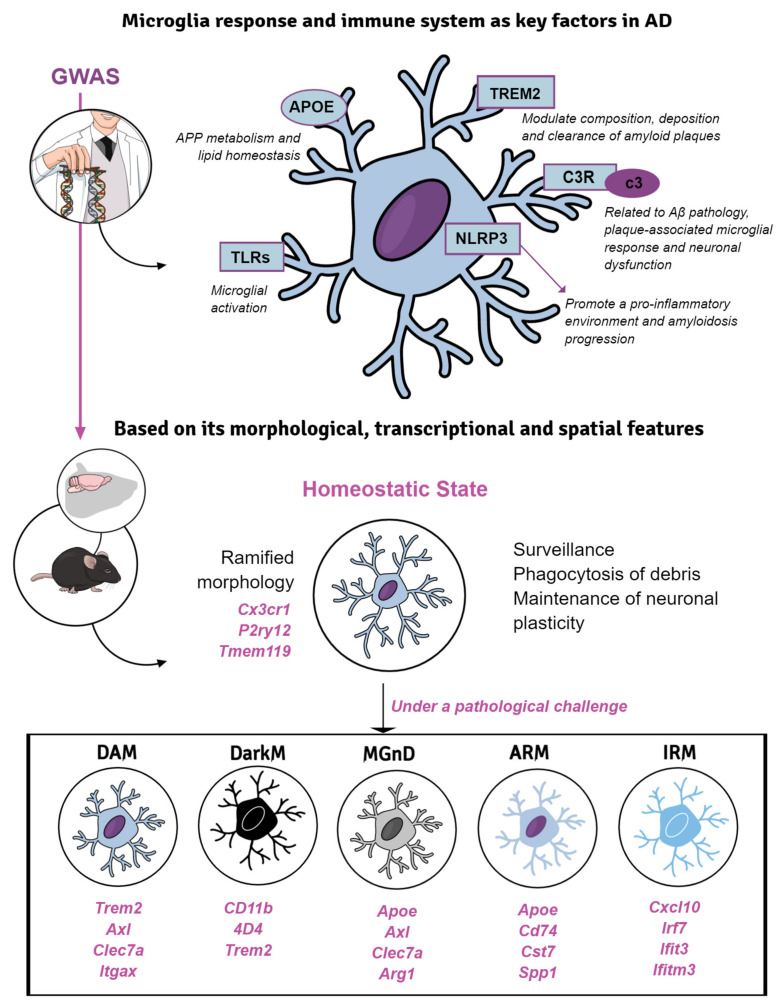
Diversity of microglial profiles identified in AD mouse models. Genome-wide association studies (GWAS) have allowed the identification of genetic risk factors associated with the development of sporadic AD. Some of them are related to the microglial immune response, such as TREM2. Transgenic mice lacking or expressing variants of proteins participating in the immune response network (TREM2, APOE, complement system, TLRs, inflammasome) have been created as tools to analyze microglial phenotypes and functions. The characterization of these models has revealed that, under pathological conditions, the homogeneity of homeostatic microglia is disrupted, giving rise to a range of dysfunctional/degenerative/activated microglial clusters that may be involved in either the onset, progression or both, of this neurodegenerative disease. APOE, apolipoprotein E; ARM, amyloid-responsive microglia; C3R, complement receptor 3; Cd74, cluster of differentiation 74; Cst7, cystatin F; Cxcl10, chemokine interferon-γ–inducible protein 10 kDa; DAM, disease-associated microglia; DarkM, dark microglia; Ifit3, interferon induced protein with tetratricopeptide repeats 3; Ifitm3, interferon induced transmembrane protein 3; Irf7, interferon regulatory factor 7; IRM, interferon-responsive microglia; MGnD, microglial neurodegenerative phenotype; NLRP3, NOD-like receptor family pyrin domain containing 3; Spp1, secreted phosphoprotein 1 or osteopontin; TLRs, Toll-like receptors; TREM2, triggering receptor expressed on myeloid cells 2.

The characterization of microglial roles in AD was initially carried out based on the first generation of FAD- or MAPT-based Tg mice. These models have allowed us to delve into the relationship between the immune system and the classical AD hallmarks. In fact, these mice have become valuable tools to investigate the variety of microglial profiles specifically involved in the progression of the AD-like pathology in vivo [210]. For instance, Aβ aggregation induces an activated microglial phenotype which cluster around fibrillar amyloid deposits. Activated microglia might play beneficial or detrimental roles to the disease pathogenesis [214]. Initially, the classification of peripheral macrophages was useful for categorizing this microglial polarization. Thus, two extreme and functionally opposite phenotypes were proposed, a pro-inflammatory or classical phenotype (M1) and a reparative or alternative phenotype (M2) [213]. However, it was not known that microglia display a surprising context-dependent heterogeneity in both healthy and diseased CNS [215,216]. According to this, microglia is currently considered a heterogeneous population that may be classified based on its morphological, transcriptional and spatial features (Figure 2). These cells are highly dynamic and quickly react adopting diverse functional states in response to injury, infection or disease challenge within the CNS [217]. Similarly, single-cell RNAseq (scRNAseq) evidenced a disease-associated microglia (DAM) state in the 5xFAD mice [218,219]. This microglial phenotype with phagocytic and neuroprotective capacity that was located in the proximity of Aβ plaques, displays upregulation of genes involved in lysosomal, phagocytic and lipid metabolism pathways and is enriched in AD-risk genes, such as Apoe, Trem2 and Tyrobp. Interestingly, a similar phenotype (microglia-associated with neurodegeneration, MGnD) has been reported in an APP/PS1 model coexpressing KM670/671NL mutated amyloid precursor protein and L166P mutated presenilin 1 under the control of a neuron-specific Thy1 promoter [220]. Both phenotypes share a decrease in the expression of homeostatic markers (Cx3cr1, P2ry12, Tmem119), an increase of phagocytic capacity and are regulated by the TREM2-APOE pathway [220,221]. In addition, dark microglia were discovered by electron microscopy in the APP/PS1dE9 model [222]. This morphological type was defined according to hyper-ramified processes, electrodense cytoplasm and nucleoplasm, mitochondrial alteration and endoplasmic reticulum dilation, and constitutes the most well-known sign of oxidative stress at the ultrastructural level. The presence of amyloid fibrils within these microglia suggests their participation in either the phagocytosis, plaque formation [217,223] or both. In fact, microglial depletion [224,225] or genetic ablation of microglial TAM receptors [51] support that fibrillar plaques are constructed by microglia. These findings have shed light on the molecular mechanisms underlying the regulation of microglial functional phenotypes in the pathogenesis of AD.

Additional transcriptomic studies have revealed the existence of other microglial states. Among them are the amyloid- and the interferon-response microglia (ARM and IRM, respectively) [226]. ARM is exclusively associated with amyloid deposits (insoluble Aβ plaques), and thus also known as PAM (plaque-associated microglia) [217,227]. ARM is characterized by its hyperreactivity in the immune response, overexpressing genes related to neuroinflammatory process (Cst7 and Clec7a), tissue repair (Dkk2, Gpnmb and Spp1) and the activation of the major histocompatibility complex (MHC) class II (Cd74, Ctsd and Ctsb) [226]. IRM, for its part, upregulates genes related to the interferon response (Cxcl10, Ifi27I2a, Irf7, Ifit3, Ifitm3), which in turn are involved in neuroinflammation and synapse loss [228,229]. However, the range of microglial states and their implication in the human pathology has yet to be fully elucidated.

The existing mouse models do not completely mimic the microglial pathology seen in AD brains. The current concept of microglial response, mostly derived from amyloidogenic models, maintains a cytotoxic and proinflammatory view, with a marked periplaque microglial reaction. However, these findings are in contrast to the limited activation and microglial degenerative state reported in the hippocampus of AD patients [230,231,232]. Microglial dysfunction may compromise the cerebral immunological protection, and therefore, be implicated in either the development, the progression, or both, of AD. Although is highly likely that AD initiates as a multifactorial process, the accumulation of toxic soluble ptau forms might be, at least in part, responsible for the microglial dysfunctional state [233]. In addition, age-related changes in cerebrovascular health, a risk factor for sAD, compromise the mitochondrial metabolism of microglial cells contributing to their dysfunctional state in AD brains [234].

Even though the relationship between tau pathology and the immunological response has been comparatively less studied, novel findings point to a strong link between both aspects. P301S mice exhibit clusters of activated microglia around tau-positive neurons, damaging dendrites and axons. These glial cells exhibited increased lysosomal volumes, showed an MGnD/DAM state and were contributing to tau propagation by phagocytosing and exocytosing tau protein [235,236]. The model rTg4510 shows a decreased expression of homeostatic genes (such as *P2ry12* and *Sall1*), associated with a more severe neuronal loss and brain atrophy, together with a strong upregulation of microglial DAM genes (such as other tau models), including *Apoe*, *Axl* and *Cybb*. These findings point to the role of APOE in the induction of DAM signature, the loss of homeostatic microglial functions, and its influence in the severity of neurodegeneration [237]. Moreover, APOE genotype influences the microglial gene profile, with APOE4 inducing a microglial proinflammatory and phagocytic phenotype in both AD models and human brains [8]. Finally, the 3xTg-AD mouse allows the simultaneous evaluation of the interplay between Aβ, tau and microglia. The results obtained from this model suggest that neuroinflammation occurs downstream of Aβ aggregation; more specifically, in association with fibrillar forms. Furthermore, microgliosis was detected at 6 months of age, preceding astroglial reactivity (at 12 months) [238].

### 5.2. Introducing AD-Risk Factors for Modeling Microglial Response in sAD

Emerging transcriptional studies based on GWAS identified variants of genes exclusively or highly expressed by microglia, which were classified as AD-risk genes. Some of them encoded for cell-surface receptors involved in the response to the pathological progression. Specifically, these genes are related to immune responses (*TREM2*, *CR1*, *CD33, MS4A*, *CLU*, *ABCA7*, *EPHA1* and *HLADRB5-HLA-DRB1*), lipid metabolism (*APOE*, *BIN1*, *PICALM*, *CD2AP*, *EPHA1* and *SORL1*) and endocytosis (*CLU*, *ABCA7* and *SORL1*). Among all of them, *TREM2* and *APOE* confer the higher risk of sAD [239,240]. For this reason, engineering new animal models by introducing these risk factors in either APP, Tau Tg mice or both, was conceived as a more accurate approach to reproduce sAD. Thus, the incorporation of novel inflammatory ingredients may yield a more appropriate recipe to gain insight into the mechanisms by which genetic-based microglial dysfunction may influence AD progression.

#### 5.2.1. TREM2

The triggering receptor expressed on myeloid cells 2 (TREM2) is a transmembrane protein abundantly expressed by microglia [241]. In 2013, genomic studies identified a rare polymorphism of this receptor resulting in the aminoacidic change R47H (rs75932628), whose odds ratio is almost as high as that associated with APOE4 (2.6–4.5-fold increased AD risk) [242,243]. Since then, other variants related to AD risk have been identified, including R62H (rs143332484), T96K (rs2234253), D87N (rs142232675) and R136Q (rs149622783). Many of them lead to a partial TREM2 loss-of-function due to a decreased affinity for its ligands [244]. Even so, additional studies are required to provide more information about the pathological mechanisms mediated by this microglial receptor. For instance, TREM2 has been proposed to modulate the composition, deposition and clearance of amyloid plaques [245]. In this regard, Aβ plaques of the TREM2-KO/5xFAD mice model were more diffuse and associated with a higher number of DNs. Therefore, silencing *Trem2* leads to an increment of amyloidosis and toxicity over neural processes, highlighting the protective role of this receptor [246,247,248,249]. Nevertheless, young TREM2-KO/APP/PS1-21 mouse model showed reduced Aβ accumulation in hippocampal areas, whereas TREM2 deficiency led to increased plaque area at later stages. These findings suggest that TREM2 may play distinct functional roles during the progression of AD pathology. In addition, TREM2 signaling is involved in regulating microglial morphology and proliferation. Similarly, microglia from TREM2-deficient mice exhibit defective capacity to surround plaques, particularly the larger ones [243,250,251]. Furthermore, microglia from TREM2-KO/APP/PS1-21 and TREM2-KO/P301S display smaller cell bodies, retraction of processes and few branches. Consequently, these alterations may impact the ability of microglia to compact and phagocyte plaques and, hence, induce local toxicity with DNs formation and the infiltration of peripheral immune cells into the brain [69]. There are conflicting results concerning the role of this receptor in neuroinflammation. Thus, TREM2-KO/P301S mice display an increase in the expression of proinflammatory cytokines including TNFα, IL-1β and IL-6 and, consequently, show a high activity of tau kinases, involved in tau hyperphosphorylation [252,253]. Conversely, TREM2-KO/APP/PS1 (4 months of age) and TREM2-KO/5xFAD mice (8.5 months) exhibit a considerable reduction in microglia-mediated neuroinflammation, expressing low levels of pro-inflammatory cytokines [247,250]. These divergences might be explained by the differences between the animal models and pathological states under analysis, and suggest a more complex role of TREM2 in neuroinflammation than previously expected [254].

#### 5.2.2. APOE, More Than a Lipoprotein

APOE is a lipoprotein implicated in APP metabolism and lipid homeostasis, being the E4 isoform the major genetic risk factor identified for sAD [8]. Besides, APOE4 serves as a ligand for several immune receptors mediating microglial activation and phagocytosis, such as TREM2 [221,255]. This lipoprotein interacts with both soluble and fibrillar Aβ, forming a complex taken-up by microglial cells in a TREM2-dependent manner. In fact, TREM2 is upregulated in plaque-associated microglia within AD brains.

Recently, two approaches have been developed to study the effects of APOE on amyloid and tau pathologies: APOE-KO, and APOE-KI mice expressing one of the three human *APOE* alleles (APOE4, APOE3 and APOE2) [8]. Either genetic deletion, haploinsufficiency or both, of *ApoE* in APP-based mouse models, such as APP/PS1-21 and APP/PS1dE9 mice, produces an increase in plaque size and DNs density, but a reduced fibrillar deposition. Moreover, microglia from these models perform an impaired response to amyloidosis, and showed a decreased expression of lipid-metabolism and phagocytosis genetic profile. These studies support a role for APOE, not only in the direct regulation of amyloid burden and plaque morphology, but also in facilitating the microglial response to amyloidosis and their ability to limit plaque-associated neuronal damage [256]. On the other hand, *APOE*-targeted replacement mice (KI) crossed with classical AD mouse models have enabled the acquisition of better knowledge about the effect of human APOE isoforms in the onset and progression of the pathology. In this context, in the 5xFAD/APOE4 and APP/PS1-21/APOE4 models, plaque burden and total Aβ42 levels were higher than in mice expressing the other isoforms. Remarkably, microglia from APOE4-KI mice exhibit a higher pro-inflammatory state and altered phagocytic function [257,258].

In relation to tau pathology, both types of models (KO and KI) have shown that microglia are the driving force in the progression of neurodegeneration in the P301S mice. P301S/APOE4 mice present higher tau levels and develop a more intense neuroinflammatory response than P301S/APOE3 and APOE2 mice, whereas the P301S/APOE-KO model was mostly protected from these changes [259,260]. All these findings suggest a promising therapeutic role for APOE to reduce amyloid and tau pathogenesis, and to modulate neuroinflammation.

#### 5.2.3. Toll-like Receptors, Complement Factors and Inflammasome

Targeting other markers related to microglial function has yielded promising results in AD mouse models. Similarly, Toll-like receptors (TLRs), a class of pattern-recognition receptors of the innate immune system involved in microglial activation, seem to perform a neuroprotective role in AD pathology [261]. The APP/PS1dE9/TLR4-KO and APP/TLR2-KO mouse models show a reduced microglial activation with low levels of IL-1β and CCL3, high amyloid burden and soluble Aβ42 levels, along with accelerated spatial and contextual memory impairments [262,263]. Furthermore, modulation of the complement pathway including C1q, C3 and C4 components produced by microglia in AD brains, may be strongly implicated in Aβ pathology, plaque-associated microglial response and neuronal dysfunction [264]. Thus, APP/PS1dE9/C3-KO mice, with a higher number of ramified microglial cells and fewer plaque-associated microglia, present reduced levels of pro-inflammatory cytokines (TNFα, IFNγ and IL-12). Apart from that, this deficiency conferred protection against synapse and neuronal loss, which translated into better learning and memory despite having more plaques due to a reduced microglial activation [265]. The model C3Ar-KO/P301S evidences the role of the complement in tau pathology. The deficiency in this receptor leads to a reduction of neurofibrillary pathology, an improvement of synaptic and neuronal function, and a decreased microglia reactivity [266].

NLRP3 inflammasome (NOD-like receptor family pyrin domain containing 3) constitutes another important immune component expressed by microglia within CNS, and can be activated by amyloid deposits, promoting a pro-inflammatory environment [267]. Recent studies in APP/PS1/NLRP3-KO mice have shown a strong reduction of Aβ deposition, correlating with a high phagocytic capacity of microglial cells for the clearance of amyloid deposits. On one hand, NLRP3 inflammasome deficiency promotes the anti-inflammatory microglial phenotype, which is associated to a higher expression of Arg-1, Il-4, FIZZ1 and insulin-degrading enzyme (IDE). On the other side, it reduces the levels of nitric oxide synthase 2 (NOS2) that accelerates the aggregation and seeding of new plaques [268]. Moreover, NLRP3 inflammasome activation has been implicated in tau pathology [269]. Altogether, these findings support the essential role of the inflammasome in the neuroinflammation as well as in the progression of AD pathology.

### 5.3. Targeting Microglial Cells in Animal Models

Ever since the contribution of microglial activation to AD pathogenesis was proposed, several approaches have been developed to deplete microglia in animal models [208,211]. One strategy is the injection of clodronate or propamidine encapsulated in liposomes into the CNS. These particles are engulfed by microglia that end up dying due to irreversible metabolic damage. Intracerebral injection of clodronate liposomes in C57BL/6 WT and CX3CR1-GFP mice elevates pro-inflammatory cytokine levels and induces astrocyte activation [270]. Although the administration of liposomes is an effective route for depleting microglia, it can damage other brain cells and compromise the integrity of blood vessels. CX3CR1, the unique fractalkine receptor expressed by microglial cells, is crucial for neuron-microglia communication [271]. CX3CR1 deficiency in AD models impacts the neuroinflammatory processes and consequently, amyloid and tau pathologies [272,273].

Global knockouts and pharmacological inhibition of genes required for microglial development/survival (TGFβ-KO, CSF1R-KO and PU.1-KO) have been widely used to induce microglia death as well [274,275]. Inhibitors (PLX3397, BLZ945, PLX5562, GW2580) of microglial cell-surface receptor CSF1R (colony stimulating factor 1 receptor) lead to improved cognition, restored spine loss, prevented neuronal death and reduced neuroinflammation in 10-month-old 5xFAD mice [276]. Additional studies have shown reduced accumulation of intraneuronal amyloid, neuritic plaques and prefibrillar oligomers in this model [224]. These findings indicate that microglia contribute to multiple facets of AD, being crucial in the onset and progression of amyloid pathology. However, these experimental manipulations not only target microglia, but also other myeloid populations (monocytes, CNS-associated macrophages, etc.) expressing these same genes. In fact, knocking out any of these genes induces severe developmental abnormalities in rodents, hindering their survival. It is important to highlight that the microglial ablation achieved in these experiments is incomplete [277]. To overcome this challenge, additional Tg lines have been developed with Cre-recombinase activity or fluorescent labeling [278]. This genetic tool has allowed manipulation and identification of microglia distinguishing them from closely related cell types [279]. Similarly, conditional genetic deletion of CSF1 receptors in microglia has now been reported [208,280] and APP/PS1/CSFR1-deleted mice ameliorated AD pathology and improved cognition [280]. Transmembrane protein 119 (Tmem119) is highly and exclusively expressed by microglial cells, so the development of TMEM119-EGFP and TMEM119-tdTomato-KI mice lines allows the discrimination of parenchymal microglia from other brain macrophages, being useful for the study of brain disorders involving monocyte infiltration, such as AD [281,282]. Another novel microglia gene-targeting model is based on the hexosaminidase subunit beta (HEXB), the lysosomal enzyme required for ganglioside GM2 degradation and a stably expressed microglial gene with no downregulation during pathology in some AD models, such as 5xFAD. Specifically, two new Tg mouse models enabling the visualization and targeting of microglia have been developed using the expression of either tdTomato or tamoxifen (TAM)-inducible expression of Cre-recombinase under the control of the endogenous Hexb promoter [283].

Despite of the fact that Tg models are constantly procuring further knowledge about the diversity of microglial phenotypes, a growing body of evidence still points out the need for their optimization to make a robust recapitulation of all the pathological AD hallmarks (amyloid plaques, neurofibrillary tangles, synaptic/neuronal loss and neuroinflammation). Nevertheless, it is undoubted that thanks to these models we are acquiring a better understanding of the molecular mechanisms involved in AD, and opening a new front in the fight against this devastating disease. In this sense, modulating neuroinflammatory response might be a promising avenue to prevent cognitive dysfunction in AD patients.

## 6. Astrocyte Reactivity in AD Brains and Models: Both Sides of the Story

Astrocytes are glial cells able to perform a broad range of homeostatic functions, including neuronal support, synaptogenesis, synaptic maintenance/pruning and blood-brain barrier (BBB) preservation, among others [284]. In addition, astroglia participate in the neuroinflammatory response triggered either by pathogens, brain injury or disease [285]. Traditionally, this cell population has received much less attention than neurons or microglia in the AD context. Fortunately, the development of techniques, such as in vivo imaging, scRNAseq and optogenetics, along with an extensive use of animal and cell models (iPSCs) have helped to shine more light onto their roles in normal and pathological conditions [286,287]. Remarkably, most of their homeostatic functions seem to be seriously affected under neuroinflammatory conditions [288,289]. In the context of AD, whether astrocyte dysfunction precedes or follows the onset of Aβ and tau pathologies remains to be determined.

### 6.1. Reactive Astrocytes in AD Brains and Models

**Figure 3:**
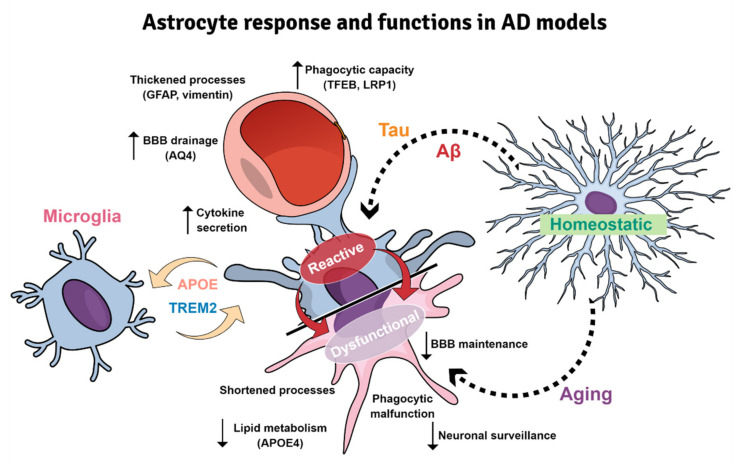
Reactive and dysfunctional astrogliosis in AD mouse models. Under pathological circumstances, such as the presence of Aβ and pathological tau, astrocytes undergo reactive astrogliosis. Reactive astrocytes are characterized by process retraction and increased thickness (overexpressing cytoskeletal proteins, such as GFAP and vimentin). They participate in the clearance of Aβ and tau by either phagocytosis or drainage through the BBB and secrete proinflammatory cytokines. On the other hand, a persistent reactive state may affect astrocytic neuronal support and BBB maintenance, induce phagocytic and lipid metabolism malfunction (especially in APOE4 genotypes), together with the shortening of their processes. Homeostatic functions of astrocytes are affected by aging as well. Reactive astrogliosis is closely connected to microglial activation in a TREM2/APOE-dependent manner. AQ4: aquaporin-4, APOE4: apolipoprotein E4; BBB: blood-brain barrier; GFAP: glial fibrillary acidic protein; LRP1: lipoprotein receptor-related protein 1; TFEB: transcription factor EB; TREM2: triggering receptor expressed on myeloid cells 2.

Astroglial cells are key regulators of the neuroinflammatory response since they can release and respond to a wide spectrum of cytokines, such as IFN*γ*, IL-1β, TNF*α*, IL-6 and TGFβ. Many of these proinflammatory molecules are upregulated in human AD brain samples and Tg mouse models [290]. Under pathological conditions, astrocytes undergo morphological and functional modifications (Figure 3), adopting a reactive phenotype. Typically, these changes include hypertrophy of the processes and cell body, along with the reorganization and polarization of their prolongations toward the site of injury. The overexpression of glial fibrillary acid protein (GFAP) and vimentin, and the re-expression of nestin [284] were considered markers of astrocyte reactivity. Other astrocyte markers, such as monoamine oxidase-B, are upregulated in neurodegenerative disorders as well [291]. At this point it is important to highlight that the concept of “astrocyte reactivity” itself is being revisited. We participated in a recent consensus study that provides a conceptual framework on astrocytes and define “reactive astrocytes” as the astrocytes undergoing “morphological, molecular, and functional changes in response to injury, disease or infection of the CNS”, thus that GFAP and other morphological markers are now insufficient to classify astrocytes as “reactive” [286]. An initial attempt to classify astrocyte response using transcriptomics yielded the LPS-like or A1 (pro-inflammatory and neurotoxic) and stroke-like or A2 (neuroprotective) profiles [292]. However, these phenotypes have not been validated in humans nor in genetically induced AD models [293]. More recent transcriptomic studies have shown that this classification oversimplifies the spectrum of astrocytic phenotypes found in AD and other neurodegenerative conditions. Recently, Habib et al. [294] identified an astrocyte population in the 5xFAD mouse model with a unique transcriptomic signature, involving high expression of GFAP along with genes related to neuroinflammation and lipid/cholesterol metabolism, which was named as disease-associated astrocyte or DAA. Future research could yield more evidence about the existence of this astrocyte state in aged or AD brains.

Despite reactive astrogliosis is not exclusive of AD, the strong relationship between this process and Aβ build-up has fostered a deeper study of astrocyte responsiveness in this specific pathological context [295]. Most APP-based mouse models display reactive astrogliosis, commonly associated with plaques. For instance, astrocyte reactivity is prominent in areas with abundant amyloid deposition in the hippocampus of the APP751SL/PS1M146L mouse model [296]. In APPswe/PS1dE9 mouse, as with many other amyloidogenic models, astrogliosis is concomitant to cognitive deterioration, showing a sexual dimorphism as described in AD patients [297].

Astrocyte reactivity may also be triggered by tau hyperphosphorylation and oligomerization, but the underlying mechanisms need to be further elucidated [298,299] In this regard, the intensity of the response to ptau differs among distinct tauopathy models. Astrocyte reactivity in the PS19 mouse strongly increases with age in the hippocampus, amygdala and entorhinal cortex, correlating with the distribution and density of NFTs [62]. In the rTgTauEC model, the number of GFAP-positive cells only increased within the entorhinal cortex (where the transgene is expressed) at later ages (14 months) [298]. Thy-Tau22 model exhibit a slight astrogliosis in the CA1 hippocampal region, the starting point of NFT pathology [65]. Although the mechanisms involved in this reaction remain obscure, recent experiments in primary cultures reported that recombinant tau can induce astrocyte response via integrin αV/β1 receptor [300]. The recently generated *App* NL-G-F/*Mapt*-KI mouse, in which the entire murine *Mapt* gene is replaced by the human *MAPT*, displays astrogliosis at 6 months of age [71]. The 3xTg-AD model presents reactive astrocytes around amyloid plaques as well [297]. The spatiotemporal characterization of astroglia in this model evidenced distinct astrocytic responses between cerebral regions, exhibiting astroglial degenerative profiles in the prefrontal and entorhinal cortices, and hippocampus [290]. Single-cell transcriptomic studies may help to clarify the reason of this diversity [301]. What is clear to date is that there is no prototypical reactive astrocyte population, nor fixed phenotypes. In fact, it has been shown that reactive astrocyte responses to tau and Aβ pathology displayed both neuroprotective and deleterious gene profiles [302].

### 6.2. Elimination of Amyloid-β and Ptau by Reactive Astrocytes: The Role of ApoE

In most amyloidogenic models, reactive astrocytes are associated to plaques, and they have been proposed to participate in Aβ elimination through degrading proteases (such as neprilysin or IDE) and phagocytosis [297]. The overexpression of the transcription factor EB (TFEB) in astrocytes from APPswe/PS1dE9 mice induced lysosome biogenesis, and enhanced the uptake of Aβ by these glial cells, not only in vitro but also in vivo [303]. Periplaque reactive astrocytes also phagocytose dystrophic presynaptic elements [296], however, Aβ is detrimental for this astrocyte phagocytic activity [304].

Interestingly, relevant AD-risk factors implicated in astrocyte function take part in brain clearance [11]. Among them, carrying the *APOE4* allele indeed constitutes the major risk factor for developing sAD [8]. The first genetic deletion of endogenous murine *ApoE* was performed in the PDAPP mice [305], revealing a critical role of this lipoprotein in amyloid accumulation and deposition. The phagocytosis and degradation of Aβ by astrocytes requires the participation of APOE, as demonstrated using APOE-null astrocytes [306]. ApoE-KO/APPswe/PS1dE9 mice presented a reduction in astrocyte reactivity and plaque burden [307]. However, the impact of APOE on reactive astrogliosis is to be defined due to important differences between mice and humans concerning this lipoprotein. Primarily, there is only one murine ApoE isoform, versus three human isoforms (E2, E3 and E4). Moreover, there exists differences in the regulatory mechanisms of APOE expression, making it more difficult to translate the results obtained with murine ApoE models [308]. That is the main reason why mice expressing human-APOE isoforms and lacking endogenous *ApoE* have become essential tools. Without any *APP*, *PS* or *MAPT* mutation, these models exhibit isoform-specific differences in lipid metabolism and synaptic function. For instance, the extent of synaptic pruning is related to APOE isoforms, since APOE4 mice show less phagocytic capacity than APOE2 or APOE3 animals [309]. However, given that these models have not yielded sufficient data to conclude that the replacement of murine ApoE with the human lipoprotein leads to AD-like pathology [297], APOE-expressing mice have been crossed with FAD Tg mice. This genetic replacement in APP-based models delayed the development of amyloid pathology. APOE4 mice displayed the highest Aβ burden compared to those expressing E2 and E3 isoforms [308]. APOE influences the uptake of Aβ mediated by low-density lipoprotein receptor-related protein 1 (LRP1) [310] as well. The expression of APOE4 in astrocytes diminished LRP1 surface expression, which may explain the impaired astrocytic amyloid clearance in vivo [311], and might also affect tau uptake. Astrocytes from APP/PS1/LRP1-KO mice also exhibited impaired clearance capacity together with amyloid accumulation [312].

On the other hand, far less is known about whether astrocytes can eliminate tau via phagocytosis or not. Perea et al. [313] reported that astrocytes can phagocyte monomeric extracellular tau. In fact, LRP1 is essential for neuronal uptake of tau, and it has been found in microglial cells and tau-positive astrocytes [314]. Interestingly, TFEB could also be implicated in tau phagocytosis and elimination, as described in the rTg4510 model by Martini-Stoica et al. [315]. Similarly, astrocyte response and ptau uptake seems to be influenced by APOE. A study comparing APOE4-KI/P301S with ApoE-KO/P301S mice found that astrocytes became more reactive in the KI model, which began to upregulate “A1” like-phenotype genes at 9 months of age. Another study with several preclinical models reported that astrocytic human-APOE enhanced the oligomerization of neuronal tau in an isoform-specific manner [316]. Shi et al. [260] noticed that APOE4 isoform induces a strong astrocyte reactivity that promotes neuronal death, whereas the absence of murine ApoE resulted in a decreased astrocytic activation and preserved neuronal integrity in vitro. Regarding the astrocytic response, the levels of some cytokines were increased in an APOE4/APPswe/PS1dE9 mouse compared to the APOE2 line [317]. Overall, these results provide more evidence that APOE4 predisposes the brain to develop AD. Additional evidence is needed to understand how APOE participates in regulating astrocyte reactivity against amyloid and tau pathologies, to determine the mechanisms involved in Aβ and tau uptake/clearance and the consequences for astrocyte integrity and functionality.

### 6.3. Clearance through the Brain Blood Barrier

Astrocytes are crucial elements in the formation and maintenance of the BBB, playing a key role in the glymphatic system [318]. The drainage of the interstitial fluid into the blood allows the clearance of substances, especially metabolic waste, and more importantly, toxic elements such as Aβ or tau. Specifically, Aβ clearance across the BBB through aquaporin-4 (AQ4) water channels is well documented [319,320]. AQ4 is highly expressed in the perivascular astrocyte foot-processes and glial limiting membrane [321]. The deletion of AQ4 exacerbates Aβ accumulation in the brain and increases memory deficits in an APP/PS1 model [322]. These data directly point to the AQP-mediated astrocyte participation in the glymphatic clearance of Aβ. Nevertheless, AQP4 was found highly diffused in the parenchyma of post-mortem AD brains and 5xFAD mouse model [323], particularly near to Aβ plaques rather than vessels, suggesting that AQP4 localization changes in AD. Interestingly, since 5xFAD mice exhibited higher levels of neuronal Aβ, AQP4 periplaque location has been proposed as a defense mechanism to counteract amyloid deposition. Further studies are needed to demonstrate this novel hypothesis [324].

APOE has been shown colocalizing with Aβ non-fibrillar forms in the perivascular space of amyloidogenic mice, suggesting the implication of this lipoprotein in Aβ clearance through the BBB [325]. Remarkably, the efficiency of APOE in binding and clearing Aβ depends on the protein isoform, being APOE4 the less efficient [326]. Thus, APPswe/PS1dE9/APOE4 mice exhibit an increase in amyloid burden and insoluble Aβ40 and Aβ42 levels, in comparison to APOE3-bearing mice [327]. The influence of APOE isoforms on the astrocyte-mediated glymphatic clearance of Aβ and tau is yet to be cleared.

Tau can be secreted from neurons in an activity-dependent manner [328], and is present in cerebrospinal fluid (CSF) and plasma [329], being considered a promising biomarker for the diagnosis of tauopathies, including AD. Nevertheless, whether astrocytes are involved in the transfer of tau through the BBB is still unknown. A recent study using a pharmacological inhibition of AQ4 activity in the rTg4510 model described a reduction in CSF-interstitial fluid exchange, clearance of tau and polarization of AQP4 [330]. However, more studies are needed to prove if these findings in murine models reflect the differences among AD patients [297].

### 6.4. Astroglial Dysfunction and Senescence: The Dark Side of Astrocyte Reactivity

As occurs with microglia, the reaction of astrocytes to brain damage or neurodegeneration constitutes an adaptive response to injury or disease. However, there is increasing evidence showing that the same pathways involved in astrocyte reactivity contribute in some degree to the loss of homeostatic functions. Thus, reactive astrogliosis might as well be translated into deleterious effects to the system as a whole. Therefore, in some circumstances, attenuation of chronic astrocyte reactivity might be beneficial [288]. Indeed, the same pathway may be protective or harmful depending on the context. For instance, STAT3-dependent transcription pathways are beneficial in traumatic brain injury (TBI) [331], but may be detrimental in an AD-context as shown in an APP/PS1 model [332].

Not only pathological conditions substantially affect glial function. Aged microglia display differences in gene expression compared to that from young individuals [333], and microglia from AD patients exhibit an accelerated aging [334]. Similarly, the affectation of astrocytic functions leads to a process called astrosenescence, defined as a reduction in astrocytic cell volume and protoplasmic processes, paralleled to changes in GFAP and vimentin expression, glutamate signaling and cholesterol metabolism dysfunction, whereas the homeostatic profile turns into a senescence-secretory phenotype [335]. However, researchers are still trying to elucidate whether or not astrocytic senescence takes place in human and mouse brains [286]. In the context of AD, it is unclear whether astrocyte degeneration/dysfunction precedes or follows the onset of either Aβ, tau or both pathologies. Similarly, Aβ has been shown to trigger astrocytic dysfunction in vitro [304], and astrocyte phagocytic response is diminished in the presence of Aβ in an APP/PS1 model [296,304]. Nevertheless, astrocyte degeneration has also been reported in the PDAPP model before Aβ deposition [336]. Astrocyte dysfunction seems to be not only restricted to loss-of-function but also including gain-of-function mechanisms. Some studies have reported that astrocytes become hyperactive in the APPKM670/671NL/PS1L166P model [337]. This calcium-dependent hyperactivity may be triggered by Aβ oligomers, thus astrocytes could in part be responsible for the early synaptic dysregulation induced by soluble Aβ oligomeric species in amyloidogenic models [338]. Sanchez-Mejias et al. [233] showed that ptau species are, at least in part, responsible for microglial senescence, whereas astrocytes remained apparently unaltered in the presence of pathological tau. Conversely, early astrocyte deficits have been detected in mouse models of tauopathy [339]. Finally, the clearance of senescent glial cells (microglia and astroglia) prevented tau pathology and improved memory decline [340] in the PS19 mice; however, the effect of senescent microglia ablation itself cannot be completely ruled out.

### 6.5. Astrocyte and Microglia Crosstalk in Mouse Models

Compelling evidence points to the existence of synchronization and communication between astrocyte and microglia, either in healthy brain or neurodegenerative conditions [341]. In fact, a strong correlation exists between both glial reactivities, that in turn parallels AD progression. Inflammatory cytokines secreted by microglia (TNFα, IL-1, C1q, among others) may alter the supporting role of astroglia [342]. Complement factors may also serve as glial intercommunicators. In the presence of Aβ oligomers, astrocyte complement factors can be secreted, and influence microglial responses in APP mouse models [343]. Likewise, activated microglia can induce astrocyte-mediated synaptic loss through the secretion of C1q, which tag synapses for their elimination. C1q has been shown to be a ligand of the astrocyte phagocytic receptor MEGF10 [344,345]. Recently, Jay et al. [346] reported that TREM2 is necessary for microglial instruction of astrocytic synaptic engulfment during neurodevelopment.

APOE is strongly upregulated in reactive microglia surrounding Aβ plaques, and has been detected in the amyloid dense-core of plaques from AD patients [347]. It has been established that APOE mediates the switch from homeostatic microglia to the DAM profile [218,220]. Moreover, APOE seems to be essential for astrocyte response and, more importantly, to communicate with microglia. According to some studies, astrocytes modulate the intensity of microglial response in either amyloidogenic [348] and tauopathy models [260] in an APOE-dependent manner. Recent work by Wang et al. [349], demonstrated that the removal of astrocytic APOE4 not only protects against tau pathology, but also decreases microglial synaptic phagocytosis in a tauopathy model. Importantly, as already mentioned, the TREM2-APOE pathway is involved in the switch of microglia to a DAM/MGnD profile (low expression of homeostatic genes and high expression of activation genes) [220,221]. TREM2-based mouse models in combination with amyloid or tau pathology strengthen the idea of microglia as a regulator of astrocytic response [297]. For instance, TREM2 deletion reduces late-stage plaque burden, Iba1 and GFAP stainings in a PS2/APP/TREM2-KO mouse model [350]. However, not every amyloid pathology induces gliosis. In a model of CAA, early-stage pathology was associated with “A1-phenotype” astrogliosis, but not with an increase in microgliosis. In fact, TREM2 levels were decreased [351]. Tg mouse models of *MAPT* and *TREM2* genes have revealed some aspects of the microglia-astrocyte response to tau pathology. In a PS19/TREM2-KO model, GFAP expression was reduced in the hippocampus at 9 months of age. Moreover, the correlation between Iba1 and GFAP-stained areas was lost compared to control PS19 mice. These results, among others [69] indicate that microglia, specifically TREM2, play a part in tau-induced astrocyte response. Additional research is needed to establish how TREM2-dependent mechanisms participate in astrocyte response in neurological disorders.

**Figure 4:**
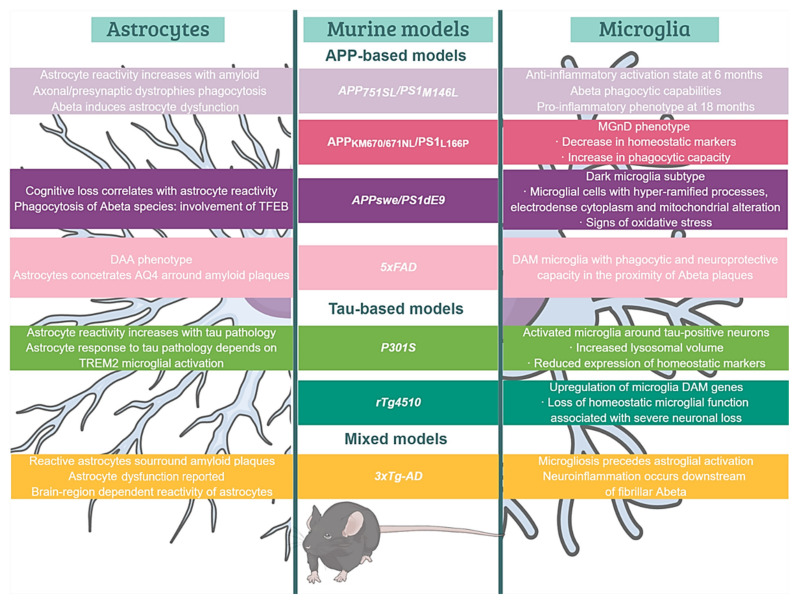
Microglial and astrocytic responses in murine models of AD. This figure summarizes some of the findings related to glial reactivity in FAD (APP-based models) and tauopathy mouse models.

Thanks to the Tg mouse models, astrocyte response may be considered a novel and promising target to focus concerning the development of new therapeutic strategies for AD patients [352]. Indeed, microglial and astroglial responses should not be considered in isolation (Figure 4). Conversely, an exhaustive study of microglial-astroglial relationship is needed, supported by the development of suitable models to unravel the mechanisms involved in the interplay of microglial activation and astrocyte reactivity. Finally, the identification of common therapeutic targets might allow the modulation of the neuroinflammatory response in a holistic way.

## 7. Oligodendrocytic Alterations in Transgenic Models of Alzheimer’s Disease

Oligodendrocytes (OLs) are highly specialized cells of the CNS. These glial cells produce myelin, the multi-layered and lipid-rich sheath that covers and insulates neuronal axons, enabling a proper and fast conduction of electrical signals along neuronal prolongations. Although white matter abnormalities have been long reported in AD brains [353], the involvement of OLs in this pathology has received little attention. In the last two years, thanks to state-of-the-art methodologies, such as scRNAseq, myelin-related alterations have been described as early and relevant events in AD pathogenesis [354,355]. Importantly, OLs respond to Aβ accumulation acquiring a reactive phenotype, similarly to what has been described for other subtypes of glial cells. These results suggest a notable and still unrevealed participation of OLs in this neurodegenerative disorder [356]. Here, we aim to gather relevant information concerning myelin and oligodendrocytic alterations in AD Tg mouse models that, on some occasions, have been described as secondary outcomes.

### 7.1. Myelin Pathology in Amyloidogenic and Tauopathy Models

Myelin disruption in AD brains was already described by Alois Alzheimer but, to date, scarce characterization of this phenomenon has been performed. Evidence exists of myelin damage in the normal aging brain, and recent data suggest that myelin alterations may influence AD progression [357], as confirmed by histological analysis of post-mortem tissue. However, our acquaintance with the importance of myelin disturbances in AD neuropathology is mainly limited to neuroimaging. Magnetic resonance imaging (MRI) studies indicate that Aβ deposition alters white matter microstructure even at the preclinical states of the disease, existing a negative correlation between myelin integrity and cognitive decline [358].

**Figure 5:**
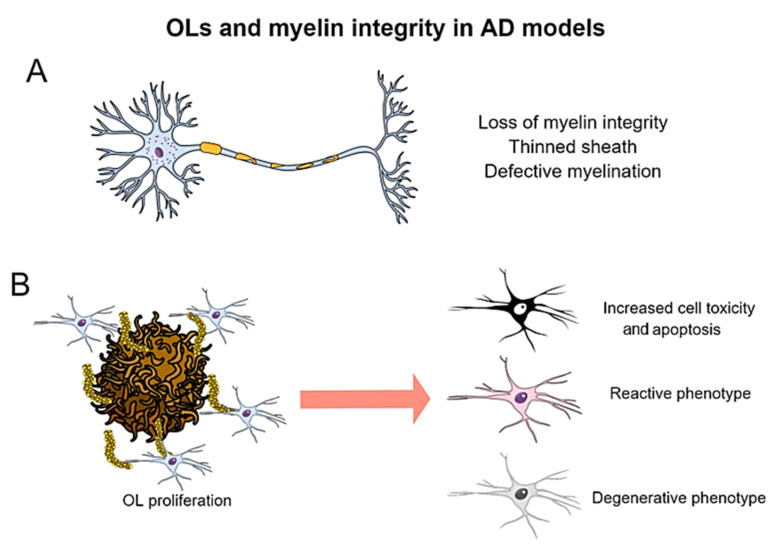
Myelin and oligodendrocyte (OL) alterations in mouse models of Alzheimer’s disease. (**A**) In AD mice, including those bearing mutations in *APP*, *PS1* or *MAPT* genes, several myelin alterations and pathologies are found from early pathological states, including loss of myelin integrity, thinned myelin sheath and signs of defective (re)myelination. (**B**) In AD mouse models, OLs have been reported to interact with both Aβ and tau pathologies. In response to this, a notable proliferation of OL progenitors occurs, in an attempt to counteract the detrimental effects that both proteinopathies induce over OLs, leading to increased cell toxicity and apoptosis. In addition, it is hypothesized that both Aβ and tau pathologies trigger a reactive and degenerative phenotype on OLSs, which redound on myelin pathology.

Notably, most of our current knowledge about myelin alterations in AD is based on studies performed in Tg murine models of this disease. Myelin deficits have been mostly reported in FAD-based mice, bearing mutations in either *APP*, *PS1* or both. In 2006, Wirths et al., described abundant age-dependent axonopathy in the spinal cord of an APP/PS1 mouse model. Myelin debris deposition, myelin ovoids and a thinned and detached myelin sheath, were indicative of nerve fiber degeneration linked to intraneuronal Aβ accumulation [359]. Further studies in the same model demonstrated that myelin alterations occurred not only in the spinal cord, but also in the brain, including focal demyelination in Aβ plaque core areas within the cortical gray matter [360], hippocampal myelin alterations, aberrant hypermyelination and decreased intermodal length [361]. Myelinic pathology, in the form of axonal swellings in the spinal cord [362] and in different cerebral regions has been also reported in the 5xFAD mouse [363,364]. Moreover, myelin thickening (another sign of altered myelin homeostasis) was observed in the hippocampus of the PDGFB-APPsweInd model [365]. Together, these findings indicate a notable myelin impairment associated to amyloid pathology (Figure 5A). Remarkably, these myelin alterations manifest early in these models, preceding plaque deposition, suggesting that OLs are influenced early during AD progression.

Even though few studies have focused on elucidating OL alterations in tauopathy models, specific myelin defects have also been detected in these mice, similarly to amyloidogenic models. For instance, rTg4510 mice (P301L mutation) exhibit unmyelinated processes at 4 months, showing disorganized patterns of myelin in different brain areas at later stages, pointing out to defective remyelination [366]. More recently, myelin disruption followed by myelin remodeling (with features of remyelination, such as myelin thinning and intermodal shortening) was reported after only 1 month of mutant tau induction in the dentate gyrus of this same model [367]. Similar myelin impairments were found in the spinal cord of the Tα1-3RT model, even preceding the emergence of tau inclusions [368]. Other studies confirmed the presence of endogenous mouse tau accumulations within OLs from rTg4510 model, suggesting that abnormal intracellular aggregation of endogenous tau in these cells might be responsible of the OL and myelin pathology of these mice [369].

Defects in myelin integrity and density have also been reported in the 3xTg-AD model from early stages [370,371,372]. These mice exhibited region-specific decline in myelination patterns, together with a reduction in the expression of the myelin basic protein (MBP) markers and 2′,3′-cyclic-nucleotide 3′-phosphodiesterase (CNPase) in the entorhinal cortex and hippocampus at 2 and 6 months, even prior to the appearance of both proteinopathies [370]. Overall, these results indicate that myelin alterations in AD brains may be associated to both amyloid and tau pathology.

### 7.2. Alterations in Oligodendroglial Proliferation and Behavior in Response to Aβ and Tau Pathologies

In addition to myelin pathology, alterations in the cells from oligodendroglial lineage have been reported in both AD brains and amyloidogenic models (Figure 5B). For instance, post-mortem studies have revealed a decrease in the number of OLs in several brain areas [373]. Conversely, other studies have described the proliferation of oligodendrocyte precursor cells (OPCs) in APP/PS1 models, especially at early time points. The number of Olig2^+^ cells increased in cortical gray matter between 6 and 9 months, concomitantly with defects in myelin [360]. Even earlier (at 2 months of age), the amount of OPCs was augmented in the hippocampus of these double Tg mice, at the same time that myelin thickening was prevalent [361]. Similarly, upregulation of NG2^+^ cells was reported in the temporal cortex of 6-month-old APPswe/PSEN1dE9 model, accompanied by the downregulation of MBP [374]. These findings suggest an early proliferation and response of OPCs in young Tg animals, probably to counteract the emerging damage to OLs and myelin sheaths. Supporting this hypothesis, an increase in new-born OLs in several brain areas (hippocampus, entorhinal cortex and fimbria) has recently been described in the PDGFB-APPsweInd model. However, total OL density remained stable, suggesting that new-born cells were only replacing the OLs lost due to disease progression [365]. In the same model, the deficiency of an important neuroprotective lipid (sphingosine 1-phosphate, S1P) that sensitizes for AD, reduced in more than 50% the hippocampal OL density. However, this marked reduction only occurred in the presence of mutated *APP*, not in controls [375].

OLs are found closely associated with Aβ pathology in APP-based models, as described in AD human brains. For instance, studies performed with the 5xFAD model described an increase in the number of OLs in response to amyloidosis. Moreover, an early and progressive up-regulation of OL genes was reported in parallel to Aβ accumulation and influenced by microgliosis [376]. In APP/PS1 mice, NG2^+^ cells not only increased in the cortex, but also clustered around amyloid plaques at 14 months of age. Moreover, these cells were able to uptake and degrade Aβ in culture, suggesting that this cell type may be involved in Aβ clearance [377]. In order to assess the influence of the microenvironment on cell fate, and to explore cell transplantation as a therapeutic possibility for AD, iPSCs were injected in the subiculum of 5xFAD at 4 months. Transplanted cells differentiated into all glial types, especially in OLs, and Aβ deposition was markedly decreased after transplantation, supporting the previous idea of a role for OLs in Aβ elimination [378].

A recent study showed plaque-associated Olig2- and NG2-expressing OPCs in the entorhinal cortex and hippocampus from 5xFAD model. These cells adopted a senescent and proinflammatory state under this environment, being unable to differentiate into myelinating OLs. Senolytic treatment of AD mice reduced plaque size, removed OPCs from Aβ deposits and ameliorated memory deficits. These results show that Aβ may induce OPCs senescence and pathology, which could be related to cognitive impairment [379]. Similarly, the number of proliferating OPCs was increased in the cortex of the APPswe/PS1dE9 mice, but they failed to differentiate into mature OLs [380]. Other groups, using the *App NL-G-F* mouse model, found a gene network enriched in myelinating factors to be activated under amyloid stress. However, this network was decreased under dense Aβ accumulation, suggesting a compensatory mechanism for OLs at the beginning of the pathology, succumbing at more advanced stages [356]. The reasons why OLs are sensitive to Aβ pathology are to be clarified. In this sense, OLs have been shown to be more vulnerable than neurons in the early pathological stages of APP (E693 mutation) model, probably mediated by endoplasmic reticulum stress [381].

Oligodendrocyte response has also been scarcely evaluated in tauopathy models. To assess whether tau pathology influenced the behavior of the oligodendrocytic population, Ossola et al. [382] performed lysolecithin-mediated focal demyelination in the spinal cord of 2-month-old P301S mice, and then analyzed the remyelinating capacity of this model. Tg mice displayed a higher OPC migration, proliferation, and maturation around and within the lesion compared to age-matched WT mice. However, this mobilization did not translate into increased remyelination since P301S-htau axons were more susceptible to demyelination-induced degeneration. In vitro studies confirmed an increased capacity of maturation in P301S-derived OPCs. Since the transgene is not expressed in the OL lineage of this model, authors concluded that the improvement in the maturation capacity may have been acquired through microenvironmental priming, thus suggesting that damaged axons signal to OPCs promoting their differentiation, in an attempt to recover myelination [382]. Performing cre-lox lineage tracing of OPCs in this same Tg model, new OLs were reported to accumulate in the hippocampus, entorhinal cortex and fimbria between 5 and 6 months of age. As the total OL density was not altered in these regions, they postulated that increased generation of OLs was accompanied by a marked oligodendroglia loss. In addition, the proportion of myelinated axons ensheathed by immature myelin was significantly higher in these areas, suggesting that newly generated and still immature OLs try to repair myelin damage present at early stages in this model [383]. These studies suggest that tau models exhibit an early myelin disruption, which may be accompanied by OPCs proliferation and differentiation with the aim of inducing remyelination. Nevertheless, this process seems to be insufficient to counteract neuronal damage.

In the 3xTg-AD model, a decline in the density of OPCs in the hippocampus was found at both 6 and 24 months. This effect was collateral to a reduction in the density and cell body shrinkage of OPC daughter cells at 6 months. In contrast, at 24 months, OPCs undergo a marked morphological hypertrophy, clustering around Aβ plaques together with astrocytes. These results demonstrate that OPCs go through complex morphological changes in 3xTg-AD mice, which are indicative of a reactive conversion [372]. Desai et al. [371] demonstrated that Aβ alters the composition of the OL pool and myelin integrity in the hippocampus at 6 months of the same model. Even though the number of Olig2^+^ OPCs remained unchanged, a significant elevation in the total number of early mature CC1^+^ OLs and a decrease in the number of MBP^+^ myelinating OLs were found. Their results indicated that amyloidosis drives myelin injury within the hippocampus of young 3xTg-AD mice, which could be due to an impaired ability of mature OLs to repair demyelinated neurons in response to Aβ [371]. Whereas mutated forms of APP and MAPT are exclusively expressed in neurons, the PS1 variant is expressed in neurons and glia, including OLs. Using a CNP-reporting 3xTg-AD mouse, this same group demonstrated that the expression of the pathogenic PS1M146V variant in OLs led to the retention of MBP within the cell body, impeding proper myelinating function by OLs. Moreover, this process is further aggravated with the exposure to pathogenic Aβ, under the influence of glycogen synthase kinase 3 beta (GSK-3β) activity [384]. Conversely, when assessed by MRI, no significant changes were found in myelin composition of white matter in 3xTg-AD mice between 11 and 17 months of age, suggesting that this approach may be not preferable to assess myelin defects, at least in this model [385].

Overall, these studies indicate that APP-based mouse models develop myelin alterations, even before plaques deposition. In addition, oligodendroglia respond to these alterations, proliferating and acquiring a reactive state, and maybe even trying to eliminate Aβ. However, new cells eventually fail to differentiate into mature OLs able to restore the myelin deficits. These facts make amyloidogenic models a suitable tool to study the role of OLs in AD. Moreover, existing studies evidence that myelin pathology emerges early in the 3xTg-AD mice, together with a notable response of the oligodendrocytic population, likely trying to counteract the myelinic damage. However, additional studies are necessary to address the influence of both Aβ and tau pathologies on OLs, and how OLs response to these two pathological events that alter brain homeostasis.

## 8. Transcriptomic and Proteomic Profiling to Assess Disease-Relevance of Mouse Models

Gene and protein expression profiling has become a powerful tool to identify the complex heterogeneity of neurons and glia under physiological and pathological conditions [386,387]. Therefore, these techniques are enabling the scientific community to classify cells into specific subtypes or states according to their unique molecular signatures, and are providing invaluable information about pathogenic pathways as well. As a result, several networks have been highlighted as the main streams involved in either the onset, pathological progression or both, of AD: proteostasis (Aβ and tau), synaptic homeostasis, inflammatory response, immunity, lipid/energy metabolism or oxidative stress [387]. The combination of techniques, together with the concomitant use of AD human and mouse samples for comparative purposes, will allow to dissect and reinforce the current knowledge about these specific pathways, and to delve into how accurate a model is to address each specific pathological event.

Wan et al. [388] performed a meta-analysis of AD cerebral transcriptomes based on more than 2000 post-mortem samples, and studied the overlaps in transcriptional alterations when comparing with 251 gen sets from 96 studies with AD mouse models. The cross-species analysis evidenced strong correspondences between neuronal/microglial genetic modules, but also pointed to some weak points in AD modeling. Their results emphasized similarities concerning responses to amyloidosis. Moreover, not only aging but also sex was revealed as an important driving factor to consider in AD research.

Spatial transcriptomics has arisen as a variant that allows studying RNA expression in tissue domains without losing positional information [389], a highly relevant factor for processes related to histopathological AD hallmarks. For instance, analyzing the vicinity of plaques (100-μm-diameter) from an AD mouse model has led to the identification of a plaque-induced genes (PIG) network involved in inflammation, the complement system, lysosomes and oxidative stress, associated to the later stages of the disease. Besides, oligodendrocyte genes (OLIGs) showed alterations at earlier phases. Importantly, most of these changes were confirmed in human brain samples [356].

At cellular level, transcriptomics has been quite relevant for understanding glial responses in AD models. As mentioned above, transcriptional scRNAseq studies allowed the identification of DAM-microglia genetic profile and the involvement of TREM2-APOE axis in the activation process [218]. Unraveling the range of microglial physiological and pathological profiles may open paths to therapeutical options specifically based on the modulation of microglial functions [219]. Similarly, recent transcriptomics is more and more evidencing the higher complexity and potential diversity of astrocytic phenotypes, including space and timing as factors to take into consideration. Das et al. [293] reported different subsets of astroglial responses depending on the context, distinguishing between chronic versus acute damage scenarios. DAA astrocytes were identified as a periplaque astrocyte population with a specific molecular signature [294] that appeared from early pathological stages in AD mice. The frequency of this astroglial subtype increased progressively during aging. Interestingly, DAA astrocytes were also found in aged WT mouse and human brains. Age-associated alterations (“age-up” and “age-down” changes) were analyzed concomitantly in both microglia and astrocytes from the APPswe/PS1dE9 mouse model by performing gene expression profiling at five time points (2, 4, 6, 9 and 12 months of age) [333]. With aging, the expression of inflammation-related genes was increased in microglia, whilst synaptic-transmission and peptidase-inhibitor genes were upregulated in astroglial cells. Interestingly, a few altered expression data were shared by these two glial populations: four age-up genes, of which three are involved in the regulation of amino-acid starvation-induced autophagy (*Cxcl10*, *Ccl2*, *Scoc)* and one in methionine salvage pathway *(Mri1*). Among the seven shared downregulated genes, three of them (*Man2b2*, *Ptbp1* and *Prrc2a*) were related to oligodendroglial specification and myelination.

Regarding tauopathy, analyzing both isolated microglia and brain tissue from Tg mice expressing mutated human *MAPT*, Rexach et al. [390] identified what they called microglial neurodegeneration-associated modules (MNMs), which overlapped with DAM microglia. In this same study, based on transcriptional network analysis of samples from mouse models and human patients of tauopathies (AD, FTD, PSP), these authors proposed the existence of microglial transitions along the AD course: initially, tau pathology triggers a proinflammatory response that is followed by an early immune suppression driven by type-1 IFN pathway, which in turn potentiates disease progression. Finally, IFN-II promotion of viral-clearance mechanisms leads to a chronic inflammation, associated to neurodegeneration. More recently, scRNAseq of microglia isolated from three different mouse models of neurodegenerative disorders (rTg4510, APP^NL-G-G/NL-G-F^ and SOD1^G93A^) evidenced that they all shared the upregulation of DAM genes. Importantly, the reduction of homeostatic genes (*P2ry12*, *Sall1*, and *Tmem119*) was mainly found in the tauopathy and motor neuron disease models, correlating with the extent of neuronal death [237].

Finally, quantitative proteomic is turning out to be another critical tool to perform wide-range comparative expression studies between animal models, and between mouse and human samples as well. In this regard, Kim et al. [391] developed a new triple Tg mouse model, ADLP^APT^, bearing six human mutations in *APP*, *PS1* and *MAPT* genes. Next, they screened and compared the hippocampal proteomes from ADLP^APT^ mice, the monogenic littermates and WT mice. They found that differentially expressed proteins (DEP) were involved in immune system and neuronal functions, and also created a protein–protein interaction map, pointing out which pathways might play interconnected or independent roles. Importantly, their database overlapped with 92% of DEP identified in human brain samples. Comparatively, “-omics” has been less addressed in tauopathy murine models, and are mainly based in FTD mice [392]. Indeed, the scientific community is taking advantage of these technologies to more quickly identify the strengths and weaknesses of animal models at mimicking AD pathology, discover novel biomarkers and detect alternative therapeutical targets.

## 9. Concluding Remarks

From the very beginning, the research with FAD-based transgenic mouse models of AD provided relevant mechanistic information to understand the basic processes underlying this type of dementia: Aβ deposition, tau pathology, synaptic damage, neuronal loss and neuroinflammation. Nevertheless, the etiology of the more prevalent sporadic AD form still remains uncertain. The tremendous complexity of this disease has become more evident with the discovery of genetic polymorphisms and comorbidities that participate, one way or another, in the onset and pathological progression. All in all, these initial results opened new horizons, leading to methodological and paradigmatic shifts so the current investigation lines can move forward using more accurate sAD models with increased translational potential. Indeed, tremendous and additional efforts are still to be made on the road to a personalized medicine, which will be based on the discovery of promising therapeutical targets able to slow the pace of this devastating disease.

## Data Availability

Not applicable.
